# Bio-Docklets: virtualization containers for single-step execution of NGS pipelines

**DOI:** 10.1093/gigascience/gix048

**Published:** 2017-06-27

**Authors:** Baekdoo Kim, Thahmina Ali, Carlos Lijeron, Enis Afgan, Konstantinos Krampis

**Affiliations:** 1 Center for Translational and Basic Research and Belfer Research Building, Hunter College of The City University of New York, 413 E 69th St, New York, NY 10021; 2 Department of Biological Sciences, Hunter College of The City University of New York, 695 Park Av., New York, NY, 10065; 3 Johns Hopkins University, Department of Biology, B3400 N Charles St, Mudd Hall 144, Baltimore MD 21218; 4 Department of Physiology and Biophysics, Institute for Computational Biomedicine, Weill Cornell Medical College, 413 E 69th St, New York, NY 10021

**Keywords:** docker, bioinformatics, NGS, RNAseq, CHIPseq

## Abstract

Processing of next-generation sequencing (NGS) data requires significant technical skills, involving installation, configuration, and execution of bioinformatics data pipelines, in addition to specialized postanalysis visualization and data mining software. In order to address some of these challenges, developers have leveraged virtualization containers toward seamless deployment of preconfigured bioinformatics software and pipelines on any computational platform. We present an approach for abstracting the complex data operations of multistep, bioinformatics pipelines for NGS data analysis. As examples, we have deployed 2 pipelines for RNA sequencing and chromatin immunoprecipitation sequencing, preconfigured within Docker virtualization containers we call Bio-Docklets. Each Bio-Docklet exposes a single data input and output endpoint and from a user perspective, running the pipelines as simply as running a single bioinformatics tool. This is achieved using a “meta-script” that automatically starts the Bio-Docklets and controls the pipeline execution through the BioBlend software library and the Galaxy Application Programming Interface. The pipeline output is postprocessed by integration with the Visual Omics Explorer framework, providing interactive data visualizations that users can access through a web browser. Our goal is to enable easy access to NGS data analysis pipelines for nonbioinformatics experts on any computing environment, whether a laboratory workstation, university computer cluster, or a cloud service provider. Beyond end users, the Bio-Docklets also enables developers to programmatically deploy and run a large number of pipeline instances for concurrent analysis of multiple datasets.

## Findings

### Background

Analysis of next-generation sequencing (NGS) data involves multiple technical steps, such as installation of the software components of bioinformatics pipelines; coordinating format conversions and data flow between pipeline components; managing software versions and updates; automating execution for multiple runs; supplying the required computational and data storage infrastructure; and last but not least, providing an intuitive user interface for nonbioinformatics experts. To overcome these challenges, bioinformatics software developers have leveraged technologies such as virtual machines and Docker containers [1, 2] for[Table tbl1] distributing preconfigured bioinformatics software that can run on any computational platform. The use of virtualization saves significant development time and cost as the software does not need to be set up from scratch at each laboratory. The increased interest for applications of virtualization for NGS data analysis is evident through many recent studies, ranging from comparing the performance of virtual machines to conventional computing [3], bioinformatics-specific Docker container repositories [4], and extensible, Docker-based bioinformatics computing frameworks [5].

The Galaxy server [6] provides an innovative approach for deployment of command-line software through an online Graphical User Interface (GUI), and it has had a great impact on making NGS data analysis tools and pipelines easily accessible to nonbioinformatics experts. In addition, the Galaxy ecosystem provides the Toolshed [7] for downloading and installing a range of commonly used bioinformatics software, with a workflow composition canvas on the GUI and a high-performance pipeline execution engine in the back end. While Galaxy workflow descriptions are standardized in eXtensible Markup Language files, allowing transfer of NGS analysis pipelines across installations at different laboratories, the bioinformatics software used in the pipelines needs to be reinstalled at each location manually or through the ToolShed. A number of different virtual machines with the Galaxy server [8] are currently available, but only 2 entries from the list include pipelines. While the virtual machines can be easily accessed with VirtualBox [9], unless users know how to set up shared folders and connect the data libraries through the Galaxy administration interface, they will have to resort to uploading large-scale datasets through the web interface, which is slow and will duplicate data within the virtual machine. Furthermore, the available Galaxy Docker containers [8] presume a level of software expertise since users need to start and log in to the containers through the command line on a local server or on the cloud.

Simpler versions of the NGS data analysis pipeline implemented in the present study have been previously published as Galaxy workflows [10, 11]. Furthermore, researchers are able to perform approximately 2 or 3 complete runs of these workflows under a single account on the public Galaxy server, given the computing time limit and storage quota of 250 GB [12], in addition to the size of NGS datasets and the amount of output generated by the bioinformatics tools composing these pipelines. Alternatively, CloudMan [13] enables users to start their own Galaxy server backed by a compute cluster on the Amazon cloud, but a number of setup steps are required [14]. In this case, researchers might be reluctant to repeatedly pay for leasing computing time and for costs associated with maintaining data on the cloud, vs a one-time investment for buying a physical computer server for their laboratory.

Besides appropriating the required compute capacity, a significant bottleneck for nonbioinformatics experts is that pipeline outputs require additional postprocessing, filtering, and visualization in order to generate scientific insights. With this in mind, our target audience is research teams that do not have any bioinformatics expertise but are generating NGS data using sequencing technology such as Illumina MiSeq or MiniSeq [15]. The Bio-Docklets approach aims to help these groups perform a basic analysis and interpretation of their datasets with minimal effort. Laboratory computers with at least 4 CPU cores and 500 GB disk storage capacity can provide enough computational capacity to run the containers with the NGS pipelines for processing the approximately 30 million reads generated per run by these instruments [16]. While the MiSeq instrument produces approximately 35 million reads, in the present study we tested our pipelines with data sets of up to 200 million reads using public datasets, and given the minimal overhead of the Docker containers by simply using a larger capacity compute server, users should be able to analyze multiples of that data size.

### Performance and testing

**Figure 1: fig1:**
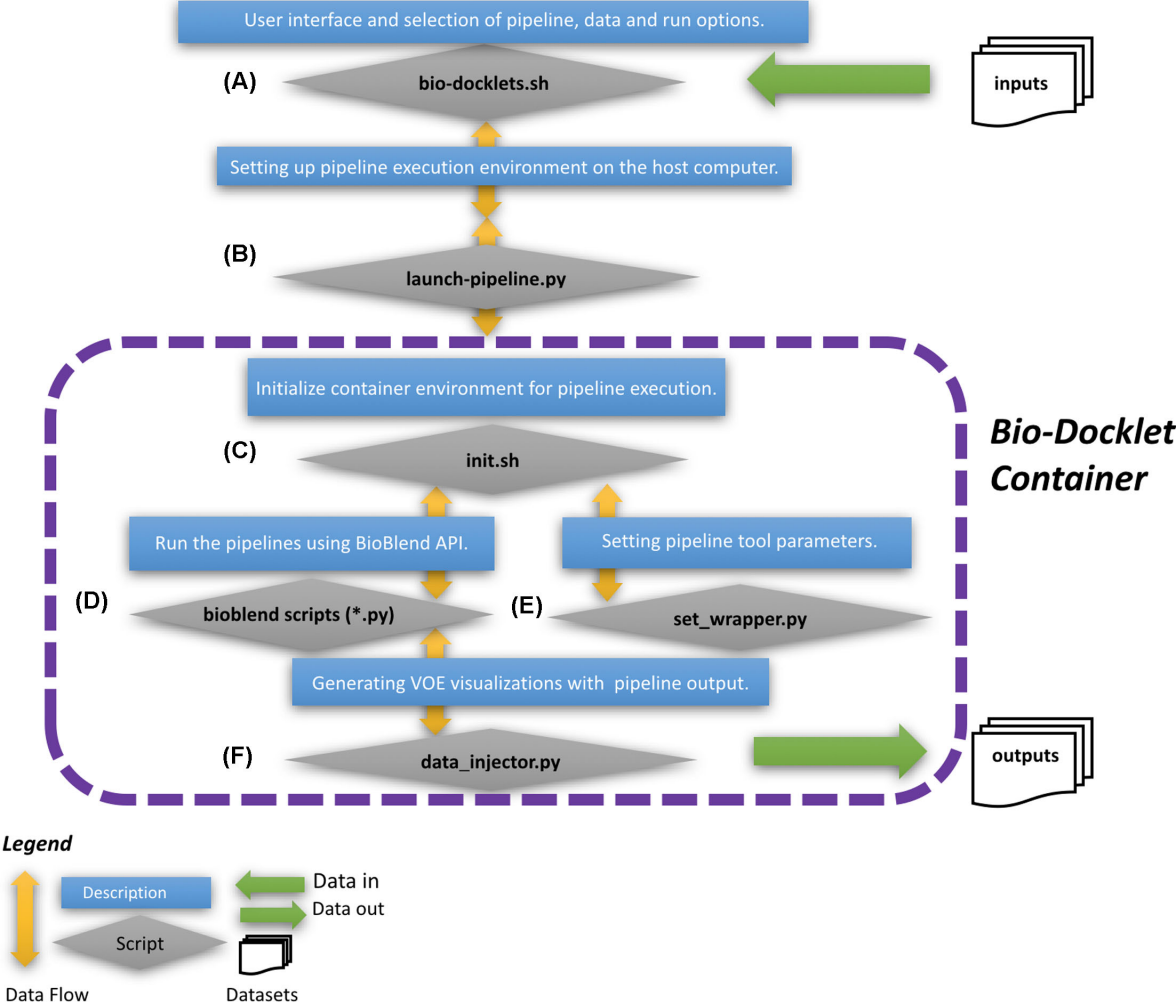
The Bio-Docklets environment with an **(a)** interactive meta-script that enables users to start the pipelines **(b)**, select analysis parameters **(c)**, and set input **(d)** and output **(e)** directories. Shell scripts and Python code were used for connecting to the Galaxy API, retrieving required data such as reference genomes, initializing environment variables in the containers, starting and monitoring the pipeline execution **(f)**. Postprocessing and loading of the pipeline output on Visual Omics Explorer interactive visualizations are saved as output in HTML/Javascript files, which can be opened on a web browser at any time after pipeline completion and container shutdown; using the visualization, the output can be mined for clusters of differentially expressed genes or histone interaction peaks, and users can export the graphics in vectorized SVG format for use in manuscripts.

In order to test the computational performance and functionality of the Bio-Docklet containers, we used publicly available NGS data from the European Bioinformatics Institute archive (EBI). First, we tested the chromatin immunoprecipitation sequencing (CHIPseq) Bio-Docklet for processing a dataset with approximately 190 million acute myeloid leukemia single-end reads and a file size of 31 GB (EBI reference ERR411994) (Table 1). The RNA sequencing (RNAseq) Bio-Docklet was tested with a 43 GB input data file (EBI reference SRR1797219 and SRR1797228) that contained a total of 188 million reads (47 million × 4, with 2 paired-end read files, for cancer and healthy tissue samples) (Table 1).[Fig fig1] We ran each Bio-Docklet in turn on our laboratory computer server (32GB RAM, 4 CPU Intel Xeon) and measured a total running time of 20 hours and 10 minutes for RNAseq to complete, while for CHIPseq the time was significantly lower, at 7 hours and 16 minutes (Table 1). This was expected, given the reduced computational capacity required for alignment of single-end reads in the CHIPseq dataset. In addition, we analyzed the same datasets with Bio-Docklets on a compute server with larger computational capacity that we rented from the Amazon Web Services cloud, and we observed a reduction of the overall compute time (Table 1). In both cases, the CHIPseq output contained the same peaks (*P* < 0.001) on chromosomes 1, 4, 5, 7, 8, 11, 16, and 19, which harbor histone interactions with an active role in tumor genesis, found in earlier studies [17], similar to RNAseq regarding the differentially expressed genes that are active regulators in cancer progression [18].

**Table 1: tbl1:** Benchmark run times of the Bio-Docklet pipeline containers with the CHIPseq and RNAseq pipelines, using as input large-scale NGS data downloaded from public databases

	CHIP-seq (total: 31 GB)	RNAseq (total: 43 GB)
Dataset location	• http://www.ebi.ac.uk/ena/data/view/ERR411994	• http://www.ebi.ac.uk/ena/data/view/SRR1797219 (cancer cells) • http://www.ebi.ac.uk/ena/data/view/SRR1797228 (healthy cells)
Dataset details	• ERR411994.fastq 192 465 714 single-end reads	• SRR1797219_1.fastq - 47 209 075 forward reads, cancer cells • SRR1797219_2.fastq - 47 209 075 reverse reads, cancer cells • SRR1797228_1.fastq - 47 697 722 forward reads, healthy cells • SRR1797228_2.fastq - 47 697 722 reverse reads, healthy cells
Running times (HH:MM:SS)		
Lab server	7:16:34	20:10:38
AWS	6:09:16	16:50:11

We have also integrated Bio-Docklets with the Galaxy CloudLaunch platform [19, 20], enabling users to acquire necessary resources from a variety of cloud providers in a few simple steps. CloudLaunch is a web portal for discovering and launching cloud-enabled applications, and it uniformly supports multiple cloud providers and multiple applications, where each application can have its user interface and launch logic tailored for the given application. For the case of Bio-Docklets, we launched a Docker-enabled virtual machine (Suppl.) that, as part of the operating system boot process, fetches the appropriate Bio-Docklet image. The wide range of instance types available from the cloud providers supported by CloudLaunch offers flexibility for users to access computational capacity at the cloud platform where they already have an account or that best fits the cost/capacity requirements, which are unique for each research group. Furthermore, Bio-Dockets can be executed by running the meta-script on any computing platform, such as the Linux servers on Amazon and Google compute clouds or a local compute server that has Docker pre-installed or that can install it during the first run of the script by providing the administrative password.

## Methods

**Figure 2: fig2:**
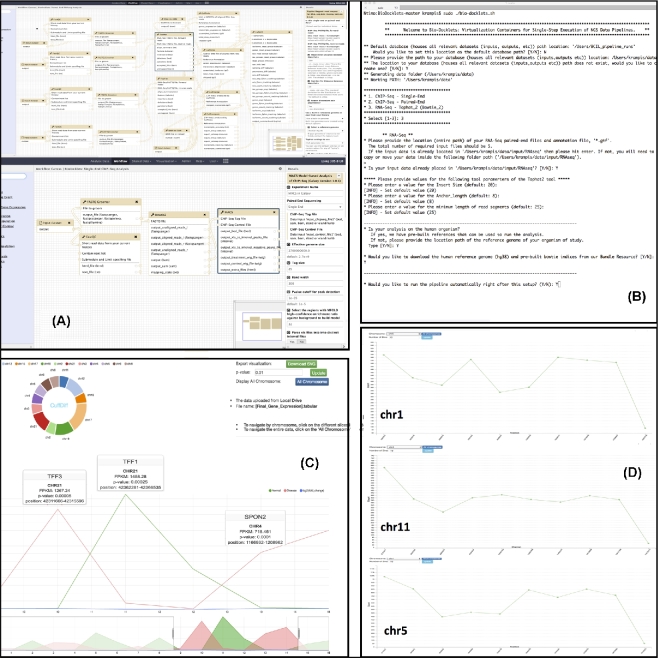
**(a)** Galaxy workflow canvas running inside the Bio-Docklets, with the composed RNAseq and CHIPseq pipelines, respectively **(b)**. User interface of the “meta-script” interactively guides the users to select which pipeline to run, input and output file directories, and reference genome indices **(c, d)**. Postprocessed pipeline output, loaded on interactive HTML/Javascript-D3 visualizations using the Visual Omics Explorer framework, can be opened in a web browser and also exported as high-resolution, manuscript-ready graphics.

In this study, we implemented the Bio-Docklets virtualization containers by combining Docker, Galaxy, and a “meta-script” (Fig. 1a), that enables users to run complex, multistep data analysis pipelines as simply as running a single bioinformatics script. In addition, we have included Python code (Fig. 1b) that leverages the BioBlend software library [21] to access the Galaxy Application Programming Interface (API), and we have automated pipeline execution using the Galaxy workflow engine running inside the container. Additional scripts implemented inside the Bio-Docklets containers (Fig. 1c,2 d, and e) automate retrieval of required datasets such as reference genomes, initialize environment parameters within the containers, and start and monitor the pipeline execution, in addition to saving all outputs to the directory specified by the user. Furthermore, we have integrated the pipelines with the Visual Omics Explorer framework (VOE) [22] through custom Python code (Fig. 1f). This code postprocesses the raw pipeline output and generates interactive HyperText Markup Language (HTML)/Javascript data visualizations that users can load on a web browser, use to perform data mining for patterns such as concentrated CHIPseq peaks or clusters of differentially expressed genes, and use to export the visualizations as publication-ready graphics. Finally, the meta-script provides details of the web address and port where the full Galaxy interface running inside the Bio-Docklets can be accessed, allowing users to use the Galaxy workflow canvas should they choose to edit the pipelines structure.

For the Bio-Docklets implementation, we started from a standard Ubuntu Linux Docker container, where we installed Galaxy and then created 2 distinct commits on DockerHub [23]. The first commit was used for implementing the RNAseq [24], and the second for the CHIPseq [25] pipeline, by first installing the bioinformatics tools used for each pipeline step from the Galaxy Toolshed if available, or manually otherwise. We then composed the pipelines through the Galaxy workflow canvas (Fig. 2a and b), and following testing, the containers were published on DockerHub. Next, we implemented a “meta-script” that automatically downloads and runs the Bio-Docklet containers from the repository, while also interactively guiding the users (Fig. 2c) to select input and output data directories and which pipeline to run, in addition to verifying the file formats and retrieving supporting data such as reference genomes. Furthermore, given administrative permissions, the script will install the Docker virtualization layer if not present on the host computing system (Suppl.). All data generated from the pipelines are saved to the output directory specified by the user, in addition to VOE visualization files in HTML5/D3.js [26] format. These files are standalone and preloaded with the pipeline output, allowing users to open them in a web browser independently of the Bio-Docklets containers and providing easy to use, interactive visualizations for data mining that can also be exported as high-resolution SVG graphics for publication.

## Discussion

Currently, a number of other bioinformatics software development projects are utilizing Docker virtualization, including, e.g., BioShaDock [4], which provides a curated repository of prebuilt bioinformatics containers, BioContainers/BioDocker [27], which implements an aggregator and search engine across Docker repositories, bioboxes [5], which defines a standardized interface for running bioinformatics tools pre-installed in containers, and Common Workflow Language (CWL) [28], which allows command line tools to be connected into portable workflows. Using the search terms “Galaxy” and “pipeline” returned 4 and 34 entries for BioShaDock and 8 and 30 for BioContainers, respectively, while bioboxes, at the time of our study, included a total of 8 containers. The BioShaDock and BioContainers repositories provide a great solution for bioinformatics developers to distribute tools and pipelines pre-installed within Docker containers and to reach the right audience, given that DockerHub is a large repository and bioinformatics containers might be missed during searches. Nonetheless, these repositories provide “Automatic Build” containers from Dockerfiles, and to the best of our efforts, we found no citations or other information on how to run the pipelines on these sites, having to resort to performing a web search to find documentation for using the tools included in the containers. Along the same lines, bioboxes provide a standardized interface where users can run bioinformatics tools and specify data directories with a single command, in addition to a novel framework for standardizing bioinformatics tool deployment in containers. While there is no user interaction or options for a workflow engine or multistep pipeline capabilities, the author of a biobox empirically preselects the appropriate parameters during implementation and, similar to Bio-Docklets abstracts, all the details from the users in order to standardize and streamline bioinformatics analysis. The CWL offers a flexible solution for composing and sharing data analysis workflows, but, for the time being at least, it is focused on the bioinformaticians composing workflows as text files—a task not aimed at biology researchers and nontechnical experts. Currently, there is no official repository of existing CWL workflows, although several instances of developed workflows can be found at online source code repositories. Importantly, those repositories are not vetted or based on pipelines published after peer review, which is the case for the pipelines made available via Bio-Docklets. Finally, executing a CWL workflow requires a CWL runtime environment on the user's system, whose setup may, again, represent a challenge for a biologist.

The NGSeasy [29] project follows a modular approach where a “master” container coordinates the pipeline run based on a workflow specification file, running “worker” containers for different bioinformatics tools for each step of the pipeline. While NGSeasy abstracts the pipeline run and coordination among the different containers, users are still required to manually install Docker and set up the required data directories, while there is no option for providing parameters for the algorithms used in the pipeline. Additional examples include GUIDock [30] and BioDepot-workflow-Builder (BwB) [31], which leverage Docker in combination with a graphical user interface. The former provides preconfigured containers for CytoScape [32], but in order to access the graphical interface, users are required to install Xquartz [33] and other specialized components, which can be challenging for nontechnical users. The BwB suite provides a pipeline composition canvas, similar to an open-source alternative to the Seven Bridges platform [34]; however, significant software development expertise is required for implementing graphical widgets and installing bioinformatics tools in separate containers. In contrast, with our approach, a researcher can easily access the rich, user-friendly interface of the Galaxy workflow canvas to easily modify or extend our pipelines through substituting the existing tools or adding new ones from the Galaxy ToolShed.

The Galaxy platform provides an option to execute containerized tools as computational jobs [35] on a local cluster or the cloud, allowing developers and system administrators to tap on the plethora of containers with preconfigured tools in order to customize and enhance the functionality of a Galaxy installation. However, this requires modifying Galaxy configuration files in addition to setting up Docker [36] on each installation separately. Our approach instead is targeted at users without the technical expertise to administer Galaxy or configure Docker, by automating the setup of both components using a single meta-script. Furthermore, our goal is to provide an integrated solution with preconfigured data analysis pipelines that can be deployed across systems ranging from single compute servers used in a laboratory to a cluster or the cloud. We realize that with the availability of Galaxy instances in Docker containers and VirtualBox machines [8], Galaxy community developers can implement solutions that provide automated deployment of all components with a similar approach to our meta-script. For example, code could be implemented that would first deploy a virtualized Galaxy server that is customized to use Docker as its job execution environment, and this code could additionally retrieve containers with bioinformatics tools from DockerHub or other repositories. This would provide a broader infrastructure deployment approach compared to ours, but would still require that developers provide a “wrapper” for new tools in order to become accessible for users through the Galaxy interface.

A significant advantage of Galaxy is scalability through the option for integration with a computer cluster in the back end [37], enabling high-throughput data analysis within a production environment. Since Bio-Docklets also include a fully featured Galaxy instance, by editing the same configuration files they can also connect to a cluster. Furthermore, on a computer server that has ample computational capacity, users can simply run the meta-script more than 1 time in order to start multiple instances of Bio-Docklets and process input datasets from different experiments in parallel. This is similar to multiple job submissions on the cluster of a typical high-throughput Galaxy installation, and despite the fact that a new Galaxy instance is started within each BioDocklet, there is minimal computational overhead since the instance runs only 1 pipeline under a single user. The Docker containers have also very little overhead, and tools such as the read aligner or transcript assembler that process millions of reads in our bioinformatics pipelines essentially consume all the computational resources. An improvement for the future would be to add to our script an option for advanced cluster integration and, through including DRMAA software libraries [38] in our containers, for the script to auto-configure these libraries for computational job submission on a specific cluster. Another approach, given that there is no limit on the resources that a Docker container utilizes, is to parallelize the pipelines internally assuming that the user has access to a powerful server to run the Bio-Docklets. While this would be feasible for tools performing independent tasks such as read alignment using, e.g., the file split options in Galaxy pipeline composition, other tools such as genome assemblers are monolithic, and the only option for scalability is if they offer the option for multithreaded execution in the implementation of the algorithm.

In our study, we have abstracted complex bioinformatics data analysis workflows in a format that is fully portable across computational platforms by encapsulating preconfigured NGS pipelines within virtualization containers we call Bio-Docklets. We leverage Galaxy as the workflow engine for coordinating execution of the software components in our pipelines and Docker as the medium for cross-platform delivery, with a focus on a specific set of pipelines that are easily accessible to users in a plug-and-play, ready-to-execute interfacing meta-script. Our goal is to enable researchers to run multistep data pipelines as simply as running as a single bioinformatics tool and perform advanced genomic data analysis without any prior technical expertise. Through the use of virtualization and the Galaxy workflow engine, the Bio-Docklets implementation essentially provides bioinformatics “black boxes” that expose a single input and output endpoint while internally performing complex bioinformatics data analysis operations. Furthermore, the BioBlend API in combination with the code included in the Bio-Docklets enables developers to programmatically manage data inputs and outputs and control the Galaxy workflow engine that runs the pipelines, in order to build bioinformatics solutions with multiple container instances for large-scale data analysis. As an alternative, we have also considered lightweight workflow engines such as NextFlow [39], but settled on Galaxy given that the ToolShed allows us to perform easy installations for some of the tools we included in the pipelines. Furthermore, access to the Galaxy server and workflow canvas running in the Bio-Docklets allows users to view and edit the pipelines from their web browser without any programming expertise. For a future update, we are working toward implementing a software platform where users can author Bio-Docklets, by composing pipelines through the Galaxy interface, and then automatically commit and publish on container repositories such as DockerHub for broad access by the community.

## Abbreviations

API: Application Programming Interface; CHIPseq: chromatin immunoprecipitation sequencing; GUI: Graphical User Interface; HTML: HyperText Markup Language; NGS: next-generation sequencing; RNAseq: RNA sequencing; VOE: Visual Omics Explorer.

## Supplementary Material

GIGA-D-17-00090_Original_Submission.pdf

GIGA-D-17-00090_Revision_1.pdf

GIGA-D-17-00090_Revision_2.pdf

Response_to_Reviewer_Comments_Original_Submission.pdf

Response_to_Reviewer_Comments_Revision_1.pdf

Reviewer_1_Report_(Original_Submission).pdf

Reviewer_2_Report_(Original_Submission).pdf

Supplemental material

## Availability of data and materials

Snapshots of the supporting code are available from the *GigaScience* repository, GigaDB [40].

## Availability of supporting source code and requirements

Project name: Bio-Docklets

Project home page: https://github.com/BCIL/BioDocklets

DockerHub: https://hub.docker.com/r/bcil/biodocklets/tags/

Operating systems: Ubuntu and MacOS

Programming language: python, shell

Other requirements: Docker virtualization layer needs to be installed for Linux or Mac

License: MIT
