# Supplementary material for: Bio-Docklets: virtualization containers for single-step execution of NGS pipelines
Source: Gigascience. 2017 Jun 27;6(8):1–7. doi: 10.1093/gigascience/gix048 (PMC5569920; doi:10.1093/gigascience/gix048)
Supplement: GIGA-D-17-00090_Original_Submission.pdf [file gix048_GIGA-D-17-00090_Original_Submission.pdf]

Manuscript
Click here to download Manuscript Bio-Docklets-Gigascience-Final-withrefs.pdf

Bio-Docklets: Virtualization Containers for Single-Step Execution of NGS Pipelines.

Baekdoo Kim[1], Thahmina Ali[1], Carlos Lijeron[1], Enis Afgan[3], and Konstantinos Krampis[1,2,4] *

[1]Center for Translational and Basic Research and Belfer Research Building, Hunter College of The City University of New York, New York; [2]Department of Biological Sciences, Hunter College of The City University of New York, New York; [3]Johns Hopkins University, Department of Biology, Baltimore, MD, [4]Department of Physiology and Biophysics, Institute for Computational Biomedicine, Weill Cornell Medical College, NY.

*Corresponding author: kk104@hunter.cuny.edu

Abstract.

Background: Processing of Next-Generation Sequencing (NGS) data requires significant technical skills, involving installation, configuration, and execution of bioinformatics data pipelines, in addition to specialized post-analysis visualization and data mining software. In order to address some of these challenges, developers have leveraged virtualization containers, towards seamless deployment of pre-configured bioinformatics software and pipelines on any computational platform.

Findings: We present an approach for abstracting the complex data operations of multi-step, bioinformatics pipelines for NGS data analysis. As examples, we have deployed two pipelines for RNAseq and CHIPseq, pre-configured within Docker virtualization containers we call Bio-Docklets. Each Bio-Docklet exposes a single data input and output endpoint and from a user perspective, running the pipelines as simple as running a single bioinformatics tool. This is achieved using a "meta-script" that automatically starts the Bio-Docklets, and controls the pipeline execution through the BioBlend software library and the Galaxy Application Programming Interface (API). The pipeline output is post-processed by integration with the Visual Omics Explorer (VOE) framework, providing interactive data visualizations that users can access through a web browser.

Conclusions: Our goal is to enable easy access to NGS data analysis pipelines for non-bioinformatics experts, on any computing environment whether a laboratory workstation, university computer cluster, or a cloud service provider. Beyond end-users, the Bio-Docklets also enables developers to programmatically deploy and run a large number of pipeline instances for concurrent analysis of multiple datasets.

Keywords

Docker, Bioinformatics, NGS, RNAseq, ChIPseq.

FINDINGS

Background

Analysis of NGS data involves multiple technical steps such as installation of the software components of bioinformatics pipelines; coordinating format conversions and data flow between pipeline components; managing software versions and updates; automating execution for multiple runs; supplying the required computational and data storage infrastructure; and last but not least, providing an intuitive user interface for non-bioinformatics experts. To overcome these challenges, bioinformatics software developers have leveraged technologies such as virtual machines and Docker containers ([1], [2]) for distributing pre-configured bioinformatics software that can run on any computational platform. The use of virtualization saves significant development time and cost, as the software does not need to be set up from scratch at each laboratory. The increased interest for applications of virtualization for NGS data analysis is evident through many recent studies, ranging from comparing performance of virtual machines to conventional computing [3], and bioinformatics-specific Docker container repositories [4].

The Galaxy server [5] provides an innovative approach for deployment of command-line software through an online Graphical User Interface (GUI), and has had a great impact on making NGS data analysis tools and pipelines easily accessible to non-bioinformatics experts. In addition, the Galaxy ecosystem provides the Toolshed [6] for downloading and installing a range of commonly used bioinformatics software, with a workflow composition canvas on the GUI and a high-performance pipeline execution engine in the back-end. While Galaxy workflow descriptions are standardized in eXtensible Markup Language (XML) files, allowing transfer of NGS analysis pipelines across installations at different laboratories, the bioinformatics software used in the pipelines need to be reinstalled at each location manually or through the ToolShed. A number of different virtual machines with the Galaxy server [7] are currently available, but only two entries from the list on [7] include pipelines. While the virtual machines can be easily accessed with VirtualBox [8], unless users know how to setup shared folders and connect the data libraries through the Galaxy administration interface, they will have to resort in uploading large-scale datasets through the web interface which is slow and will duplicate data within the virtual machine. Furthermore, the available Galaxy Docker containers [7] presume a level of software expertise, since users need to start and login to the containers through the command line on a local server or on the cloud.

Simpler versions of the NGS data analysis pipeline implemented in the present study, have been previously published as a Galaxy workflows [9,10]. Furthermore, researchers are able to perform approximately two or three complete runs of these workflows under a single account on the public Galaxy server, given the computing time limit and storage quota of 250GB [11], in addition to the size of NGS datasets and the amount of output generated by the bioinformatics tools composing these pipelines. Alternatively, CloudMan [12] enables users to start their own Galaxy server backed by a compute cluster on the Amazon cloud, but a number of setup steps are required [13]. In this case, researchers might be reluctant to repeatedly pay for leasing computing time and for costs associated with maintaining data on the cloud, versus a one-time investment for buying a physical computer server for their laboratory.

Besides appropriating the required compute capacity, a significant bottleneck for non-bioinformatics experts is that pipeline outputs require additional post-processing, filtering and visualization in order to

generate scientific insights. With this in mind, our target audience is research teams that do not have any bioinformatics expertise, but are generating NGS data using sequencing technology such as Illumina MiSeq or MiniSeq [14]. The Bio-Docklets approach aims to help these groups perform a basic analysis and interpretation of their datasets with minimal effort. Laboratory computers with at least 4 CPU cores and 500GB disk storage capacity, can provide enough computational capacity to run the containers with the NGS pipelines for processing the approximately 30 million reads generated per run by these instruments [15]. Furthermore, in the present study we demonstrate that researchers can use the containers to scale up the analysis up to 200 million reads, with a larger capacity server.

Performance and testing

In order to test the computational performance and functionality of the Bio-Docklet containers, we used publicly available NGS data from the European Bioinformatics Institute archive (EBI). First, we tested the CHIPseq Bio-Docklet for processing a dataset with approximately 190 million Acute Myeloid Leukemia (AML) single-end reads, and file size 31GB (EBI reference ERR411994, **Table 1**). The RNAseq Bio-Docklet was tested with a 43GB input data file (EBI reference SRR1797219 and SRR1797228), that contained a total of 188 million reads (47 million x 4, with two paired-end read files, for cancer and healthy tissue samples, **Table 1**). We run each Bio-Docklet in turn on our laboratory computer server (32GB RAM, 4 CPU Intel Xeon), and measured a total running time of 20 hours and 10 minutes for RNAseq to complete, while for CHIPseq the time was significantly lower at 7 hours and 16 minutes (**Table 1**). This was expected, given the reduced computational capacity required for alignment of single-end reads in the CHIPseq dataset. In addition, we analyzed the same datasets with Bio-Docklets on a compute server with larger computational capacity that we rented from the Amazon cloud (AWS) and we observed reduction of the overall compute time (**Table 1**). In both cases, for the CHIPseq output we observed a large number of significant peaks (p-value < .001) on chromosomes 1, 4, 5, 7, 8, 11, 16 and 19, which harbor histone interactions with active role to tumor genesis [16], while for RNAseq we found differentially expressed genes that are active regulators in cancer progression [17].

Beyond availability for local computer servers and the Amazon cloud, we have also integrated Bio-Docklets with the Galaxy CloudLaunch platform [18,19], enabling users anyone to acquire necessary resources from a variery of cloud providers in a few simple steps. CloudLaunch is a web portal for discovering and launching cloud-enabled applications, and uniformly supports multiple cloud providers and multiple applications where each application can have its user interface and launch logic tailored for the given application. For the case of Bio-Docklets, we launched a Docker-enabled virtual machine (Suppl.) and, as part of the operating system boot process, fetch the appropriate Bio-Docklet image. With the wide range of instance types available from the cloud providers supported by CloudLaunch, this offers flexibility for users to access computational capacity at the cloud platform they already have an account or fits best the cost/ capacity requirements, unique for each research group.

Table 1: Benchmark run times of the Bio-Docklet pipeline containers with the ChIPseq and RNAseq pipelines, using as input large-scale NGS data downloaded from public databases.

	**ChIP-Seq (total: 31 GB)**	**RNA-Seq (total: 43 GB)**
**Dataset Location**	://www.ebi.ac.uk/ena/data/view/ERR4119	://www.ebi.ac.uk/ena/data/view/SRR17972 (cancer cells)<br><br>://www.ebi.ac.uk/ena/data/view/SRR17972 (healthy cells)
**Dataset Details**	R411994.fastq 192,465,714 single-end ds	R1797219_1.fastq - 47,209,075 forward ds, cancer cells<br><br>R1797219_2.fastq - 47,209,075 reverse ds, cancer cells<br><br>R1797228_1.fastq - 47,697,722 forward ds, healthy cells<br><br>R1797228_2.fastq - 47,697,722 reverse ds, healthy cells
**Running Times (HH:MM:SS)**		
**Lab Server**	7:16:34	20:10:38
**AWS**<br><br>http://aws.amazon.com	6:09:16	16:50:11

METHODS

We implemented the Bio-Docklets virtualization containers by combining Docker, Galaxy, and a "meta-script" (**Fig. 1a**), that enables users to run complex, multi-step data analysis pipelines, as simply as running a single bioinformatics script. In addition, we have included Python code (**Fig. 1b**) that leverages the BioBlend software library [20] to access the Galaxy API, and automate pipeline execution using the Galaxy workflow engine running inside the container. Additional scripts implemented inside the Bio-Docklets containers (**Fig. 1c,d,e**) automate retrieval of required datasets such as reference genomes, initialize environment parameters within the containers, start and monitor the pipeline execution, in addition to saving all outputs to the directory specified by the user. Furthermore, we have integrated the pipelines with the Visual Omics Explorer framework (VOE, [21]) through custom Python code (**Fig 1f**). This code post-processes the raw pipeline output and generates interactive HTML / Javascript data visualizations that users can load on a web browser, perform data mining for patterns such as concentrated CHIPseq peaks or clusters of differentially expressed genes, and export the visualizations as publication-ready graphics. Finally, the meta-script provides details of the web address and port where the full Galaxy interface running inside the Bio-Docklets can be accessed, allowing users to use the Galaxy workflow canvas should they choose to edit the pipelines structure.

For the Bio-Docklets implementation we started from a standard Ubuntu Linux Docker container, where we installed Galaxy and created two distinct commits on DockerHub [22]. The first commit was used for implementing the RNAseq [23] and the second for the CHIPseq [24] pipeline, by first installing the bioinformatics tools used for each pipeline step from the Galaxy Toolshed if available, or manually otherwise. We then composed the pipelines through the Galaxy workflow canvas (**Fig. 2a, 2b**), and following testing the containers were published on DockerHub. Next, we implemented a "meta-script" that automatically downloads and runs the Bio-Docklet containers from the repository, while also interactively guides the users (**Fig 2c**) to select input and output data directories, which pipeline to run, in addition to verifying the file formats and retrieving supporting data such as reference genomes. Furthermore, given administrative permissions the script will install the Docker virtualization layer if not present on the host computing system (**Suppl.**). All data generated from the pipelines are saved to the output directory specified by the user, in addition to VOE visualization files in HTML / Javascript-D3 [25] format. These files are standalone and pre-loaded with the pipeline output, allowing users to open them in a web browser independently of the Bio-Docklets containers, and providing easy to use, interactive visualizations for data mining that can also be exported as high-resolution SVG graphics for publication.

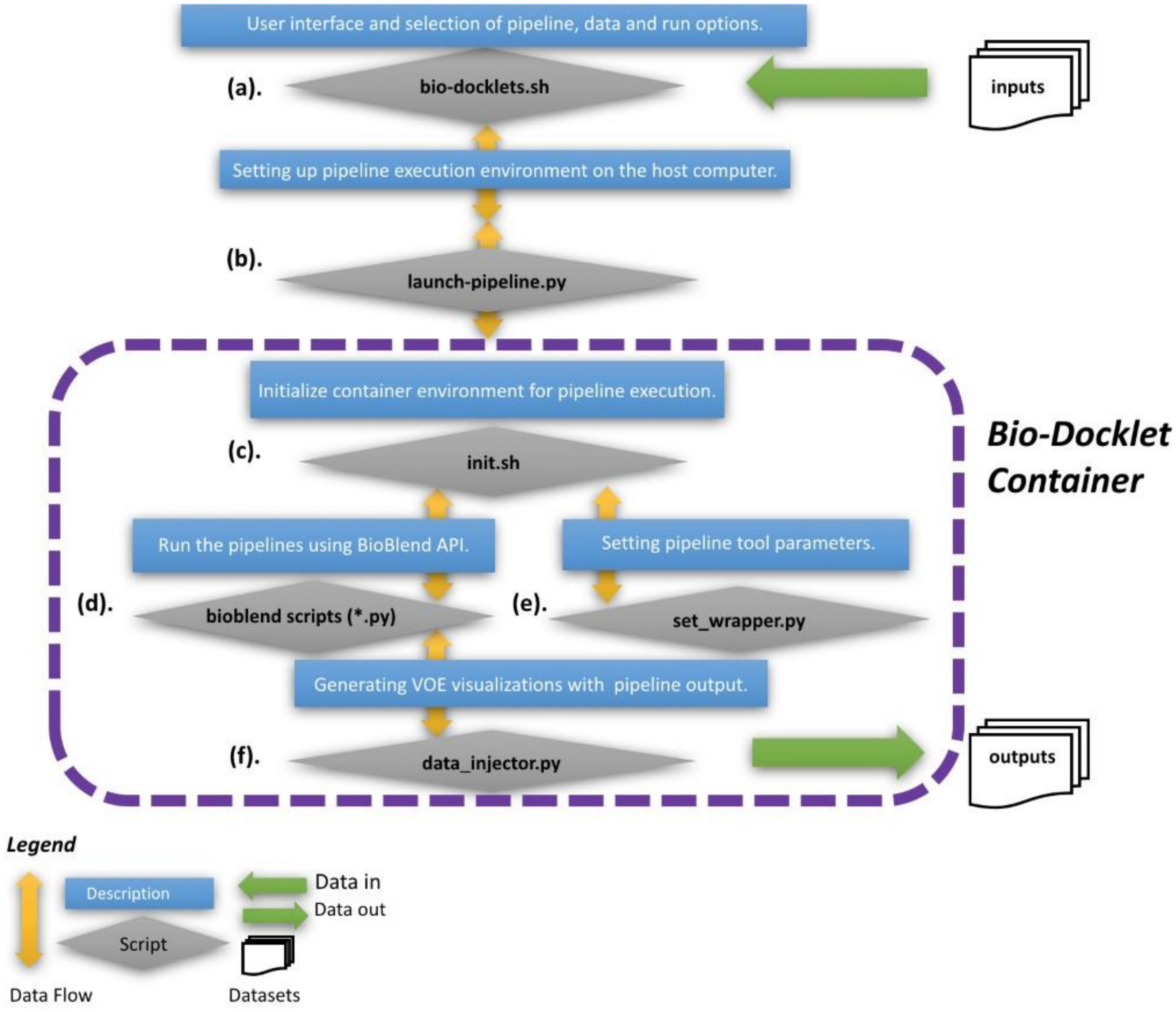

Fig. 1. The Bio-Docklets environment with an **(a).** Interactive meta-script that enables users to start the pipelines, select analysis parameters, and set input and output directories; **(b, c, d, e).** Shell scripts and Python code used for connecting to the Galaxy API, retrieving required data such as reference genomes, initializing environment variables in the containers, starting and monitoring the pipeline execution; **(f).** post-processing and loading of the pipeline output on Visual Omics Explorer (VOE) interactive visualizations that are saved as output in HTML / Javascript files, which can be opened on a web browser at any time after pipeline completion and container shutdown; using the visualization the output can be mined for cluster of differentially expressed genes or histone interaction peaks, and users can export the graphics in high-resolution SVG format for use in manuscripts.

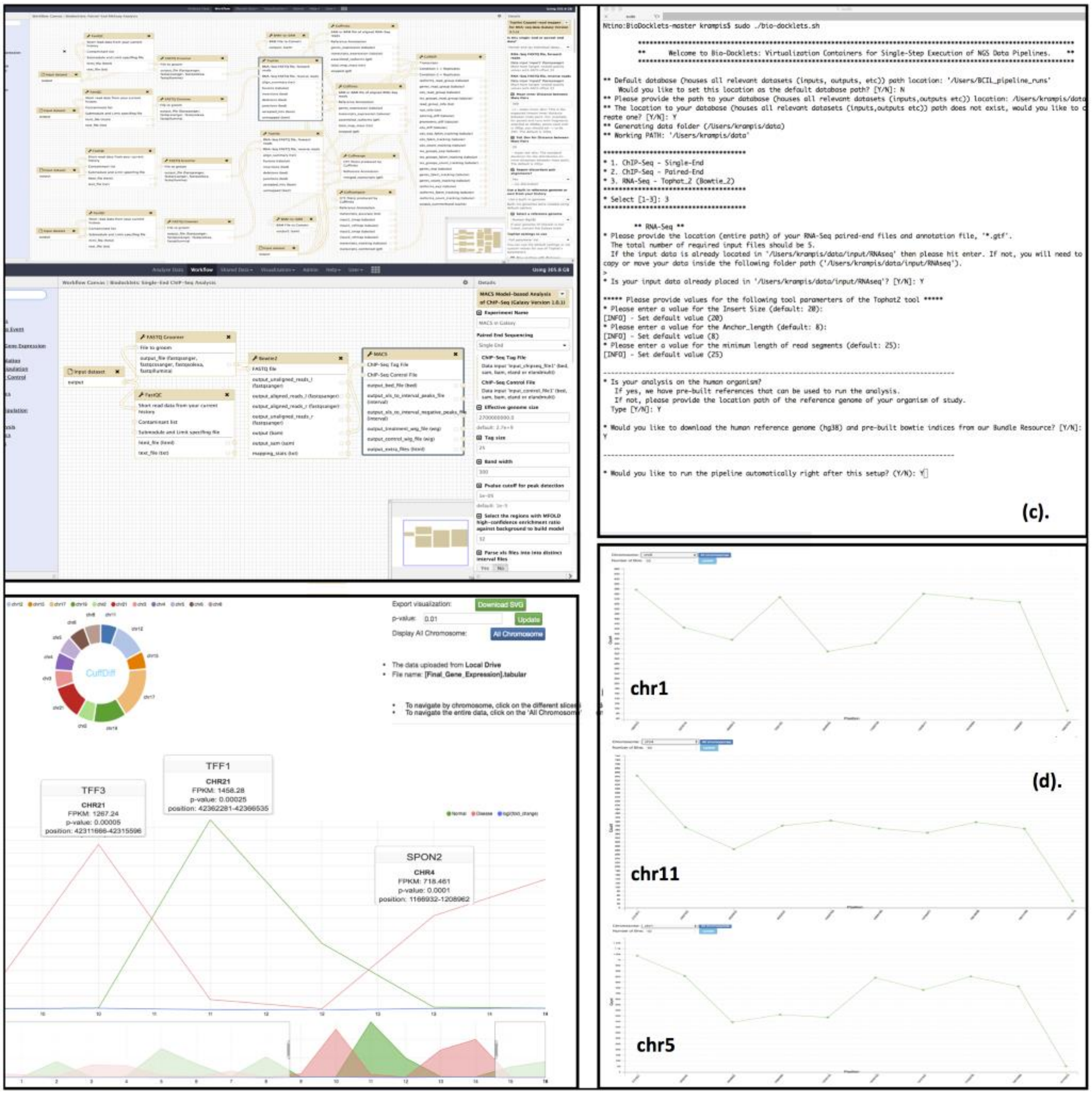

Fig. 2. **(a,b).** Galaxy workflow canvas running inside the Bio-Docklets, with the composed RNAseq and CHIPseq pipelines respectively; **(b).** User interface of the "meta-scipt", that interactively guides the users to select which pipeline to run, input and output file directories and reference genome indices; **(c).** Post-processed pipeline output, loaded on interactive HTML / Javascript-D3 visualizations using the Visual Omics Explorer framework that can be opened in a web browser and also exported as high-resolutions, manuscript-ready graphics.

DISCUSSION

Currently, a number of bioinformatics projects are utilizing Docker virtualization, including for example BioShaDock [4] that provides a curated repository of pre-built bioinformatics containers, BioContainers / BioDocker [26] that implements an aggregator and search engine across Docker repositories, and BioBoxes [27] that defines a standardized interface for running bioinformatics tools pre-installed in containers. Using the search terms “Galaxy” and “pipeline” returned 4 and 34 entries for BioShaDock, 8 and 30 for BioContainers respectively, while BioBoxes at the time of our study included a total number of 8 containers. The BioShaDock and BioContainers repositories, provide a great solution for bioinformatics developers to distribute tools and pipelines pre-installed within Docker containers and to reach the right audience, given that DockerHub is a large repository and bioinformatics containers might be missed during searches. Nonetheless these repositories provide “Automatic Build” containers from Dockerfiles, and to the best of our efforts we found no citations or other information how to run the pipelines on theses sites, having to resort to performing a web search to find documentation for using the tools included in the containers. Along the same lines, BioBoxes provide a standardized interface where users can run bioinformatics tools and specify data directories with a single command, but no user interaction or other options for using the containers are provided. While BioBoxes introduces a novel framework for standardizing bioinformatics tool deployment in containers, to the best of our knowledge this framework does not include a workflow engine or multi-step pipeline execution capabilities.

The NGSeasy [28] project follows a modular approach where a “master” container coordinates the pipeline run based on a workflow specification file, running "worker" containers for different bioinformatics tools for each step of the pipeline. While NGSeasy abstracts the pipeline run and coordination among the different containers, users are still required to manually install Docker and setup the required data directories, while there is no option for providing parameters for the algorithms used in the pipeline. Additional examples include GUIDock [29] and BioDepot-workflow-Builder (BwB, [30]), that leverage Docker in combination with a graphical user interface. The former provides pre-configured containers for CytoScape [31], but in order to access the graphical interface users are required to install Xquartz [32] and other specialized components, which can be challenging for non-technical users. The BwB suite provides a pipeline composition canvas, similar to an open-source alternative of the Seven Bridges platform [33], however significant software development expertise is required for implementing graphical widgets and installing bioinformatics tools in separate containers. In contrast, with our approach a researcher can easily access the rich, user-freindly Galaxy interface, and should they require to modify the pipelines to download and install additional tools from the extensive list of the ToolShed without any software development effort.

In our study, we have abstracted complex bioinformatics data analysis workflows in a format that is fully portable across computational platforms, by encapsulating pre-configured NGS pipelines within virtualization containers we call Bio-Docklets. Our goal is to enable researchers to run multi-step data pipelines as simply as running as a single bioinformatics tool, and perform advanced genomic data analysis without any prior technical expertise. Through the use of virtualization and the Galaxy workflow engine, the Bio-docklets implementation essentially provides bioinformatics “black-boxes” that expose a single input and output endpoint, while internally perform complex bioinformatics data analysis operations.

Furthermore, the BioBlend API in combination with the code included in the Bio-Docklets enables developers to programmatically manage data inputs, output, and control the Galaxy workflow engine that runs the pipelines, in order to build bioinformatics solutions with multiple container instances for large-scale data analysis. As an alternative, we have also considered lightweight workflow engines such as NextFlow [34], but settled on Galaxy given that the ToolShed allows us to perform easy installations for some of the tools we included in the pipelines. Furthermore, access to the Galaxy server and workflow canvas running in the Bio-Docklets, allows users to view and edit the pipelines from their web browser without any programming expertise. For a future update, we are working towards implementing a software platform where users can author Bio-Docklets by composing pipelines through the Galaxy interface, and then automatically commit and publish on container repositories such as DockerHub for broad access by the community.

Declarations

Ethics (and consent to participate)

Not applicable

Consent to publish

Not applicable

Competing interests

Not applicable

Authors' contributions

BK developed all automated script and constructed method architecture. TA implemented method pipelines, performed data analysis and method validation. CL assisted with method validation and manuscript preparation. KK wrote the manuscript, supervised all the work from conception to manuscript preparation and review.

Availability of data and materials

Bio-Docklets containers repository on DockerHub: https://hub.docker.com/r/bcil/biodocklets/tags/

Bio-Docklets meta-script: https://github.com/BCIL/BioDocklets

List of abbreviations used (if any)

NGS Next Generation Sequencing **GUI** Graphical User Interface **XML** eXtensible Markup Language **RNAseq** RNA sequencing **ChIPseq** Chromatin Immunoprecipitation sequencing **AWS** Amazon Web Services **HTML** HyperText Markup Language **VOE** Visual Omics Explorer **USB** Universal Serial Bus **API** Application Programming Interface

Acknowledgements

Funding: Supported by the CTBR and RCMI grant from NIMHD (G12 MD007599) and WCMC-CTSC (2UL1TR000457-06). The authors would like to thank all members of the Bioinformatics Core Infrastructures and Krampis' Lab for their feedback during manuscript preparation.

1. Krampis K, Booth T, Chapman B, Tiwari B, Bicak M, Field D, et al. Cloud BioLinux: pre-configured and on-demand bioinformatics computing for the genomics community. BMC Bioinformatics 2012;13:1–8.

2. Hosny A, Vera-Licona P, Laubenbacher R, Favre T. AlgoRun: A Docker-based packaging system for platform-agnostic implemented algorithms. Bioinformatics. 2016;32:2396–8.

3. Di Tommaso P, Palumbo E, Chatzou M, Prieto P, Heuer ML, Notredame C. The impact of Docker containers on the performance of genomic pipelines. PeerJ [Internet]. 2015;3:e1273.

4. Moreews F, Sallou O, Ménager H, Le bras Y, Monjeaud C, Blanchet C, et al. BioShaDock: a community driven bioinformatics shared Docker-based tools registry. F1000Research [Internet]. 2015;1–9.

5. Goecks J, Nekrutenko A, Taylor J. Galaxy: a comprehensive approach for supporting accessible, reproducible, and transparent computational research in the life sciences. Genome Biol. 2010;11:R86.

6. Blankenberg D, Von Kuster G, Bouvier E, Baker D, Afgan E, Stoler N, et al. Dissemination of scientific software with Galaxy ToolShed. Genome Biol. 2014;15:403.

7. List of Galaxy Virtual Appliances: https://wiki.galaxyproject.org/VirtualAppliances.

8. VirtualBox software: http://www.virtualbox.org

9. Galaxy RNAseq published workflow: https://usegalaxy.org/u/fluidigmngs/w/rnaseq-workflow

10. Galaxy CHIPseq published workflow: https://usegalaxy.org/u/chip-seq-helin-group/w/mmusculus-mm10-create-bam-bigwig-and-peakcalling-for-chip-seq

11. Public Galaxy server quotas: https://wiki.galaxyproject.org/Main#User_Account_Quotas

12. Afgan E, Chapman B, Taylor J. CloudMan as a platform for tool, data, and analysis distribution. BMC bioinformatics. 2012 Nov 27;13(1):315.

13. Starting a Galaxy cluster on the Amazon Web Services (AWS) with CloudMan: https://wiki.galaxyproject.org/CloudMan/AWS/GettingStarted

14. Illumina MiniSeq sequencing technology: http://www.illumina.com/systems/miniseq.html

15. Illumina MiniSeq sequencing output: https://www.illumina.com/systems/miseq/kits.html.html

16. Zhang KQ, Salzman SA, Reding DJ, Suarez BK, Catalona WJ, Burmester JK. Genetics of prostate cancer. Clin Med Res. 2003;1:21–8.

17. Huang KH, Chow KC, Chang HW, Lin TY, Lee MC. ATPase family AAA domain containing 3A is an anti-apoptotic factor and a secretion regulator of PSA in prostate cancer. Int. J. Mol. Med. 2011;28:9–15.

18. Galaxy CloudLaunch : http://launch.usegalagy.org

19. Afgan E, Krampis K, Goonasekera N, Skala K, Taylor J. Building and provisioning bioinformatics environments on public and private clouds. InInformation and Communication Technology, Electronics and Microelectronics (MIPRO), 2015 38th International Convention on 2015 May 25 (pp. 223-228). IEEE.

20. Leo S, Pireddu L, Cuccuru G, Lianas L, Soranzo N, Afgan E, et al. BioBlend.objects: Metacomputing with galaxy. Bioinformatics. 2014;30:2816–7.

21. Kim B, Ali T, Hosmer S, Krampis K. Visual Omics Explorer (VOE): a Cross-Platform Portal for Interactive Data Visualization. Bioinformatics [Internet]. 2016;32:btw119.

22. Bio-Docklets container repository on DockerHub: https://hub.docker.com/r/bcil/biodocklets/

23. Trapnell C, Roberts A, Goff L, Pertea G, Kim D, Kelley DR, et al. Differential gene and transcript expression analysis of RNA-seq experiments with TopHat and Cufflinks. Nat. Protoc. [Internet]. 2012;7:562–78.

24. Zhang Y, Liu T, Meyer CA, Eeckhoute J, Johnson DS, Bernstein BE, et al. Model-based analysis of ChIP-Seq (MACS). Genome Biol. 2008;9:R137.

25. The Javascript-D3 programming library: https://d3js.org

26. BioDocker: An open-source and community-driven framework for software standardization by Felipe Da Veiga Leprevost - Research Project on ResearchGate [Internet]. [cited 2017 Feb 6].

27. Belmann P, Dröge J, Bremges A, McHardy AC, Sczyrba A, Barton MD. Bioboxes: standardised containers for interchangeable bioinformatics software. Gigascience [Internet]. 2015;4:47.

28. Folarin AA, Dobson RJ, Newhouse SJ, Folarin AA, Dobson RJ, Newhouse SJ. NGSeasy: a next generation sequencing pipeline in Docker containers. F1000Research 2015 [cited 2017 Feb 5];4

29. Hung LH, Kristiyanto SB, Yeung KY (2016). "GUIdock: Using Docker Containers with a Common Graphics User Interface to Address the Reproducibility of Research." PLoS One **11**(4): e0152686.

30. Hung LH, Meiss T, Keswani J, Xiong Y, Sobie E, Yeung KY. Building containerized workflows for RNA-seq data using the BioDepot-workflow-Builder (BwB). bioRxiv. 2017 Jan 1:099010.

31. Cytoscape biological network visualization tool: http://www.cytoscape.org

32. Xquarz graphics engine: http:// www.xquartz.org

33. Seven Bridges cloud platform: https://www.sevenbridges.com/platform/

34. Nextflow bioinformatics workflow language : http://www.nextflow.io

Figure 1

Click here to download Figure Bio-docklets_figure1-GigaScience.jpg

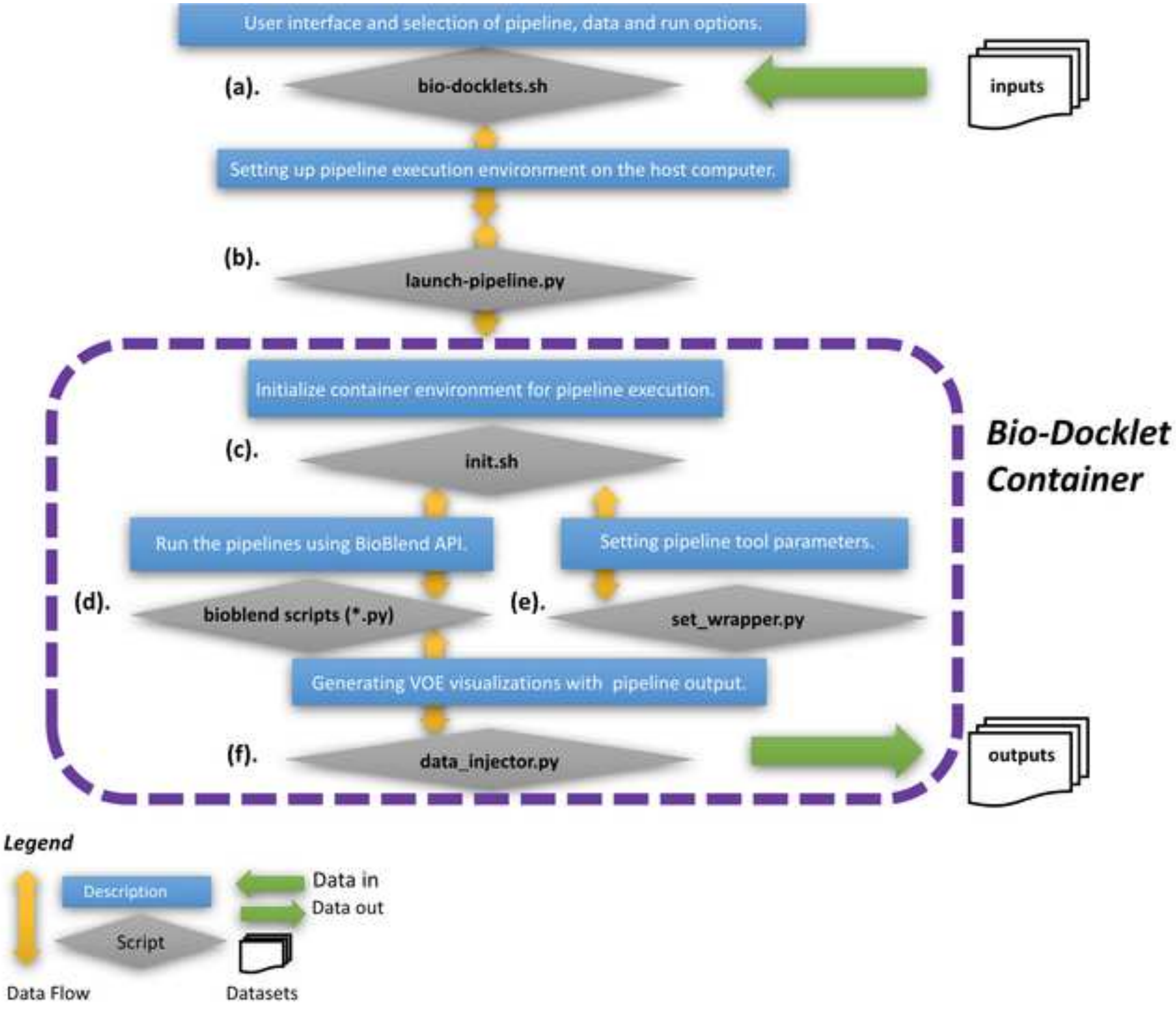

Figure 2

Click here to download Figure Bio-docklets_figure2-GigaScience.png

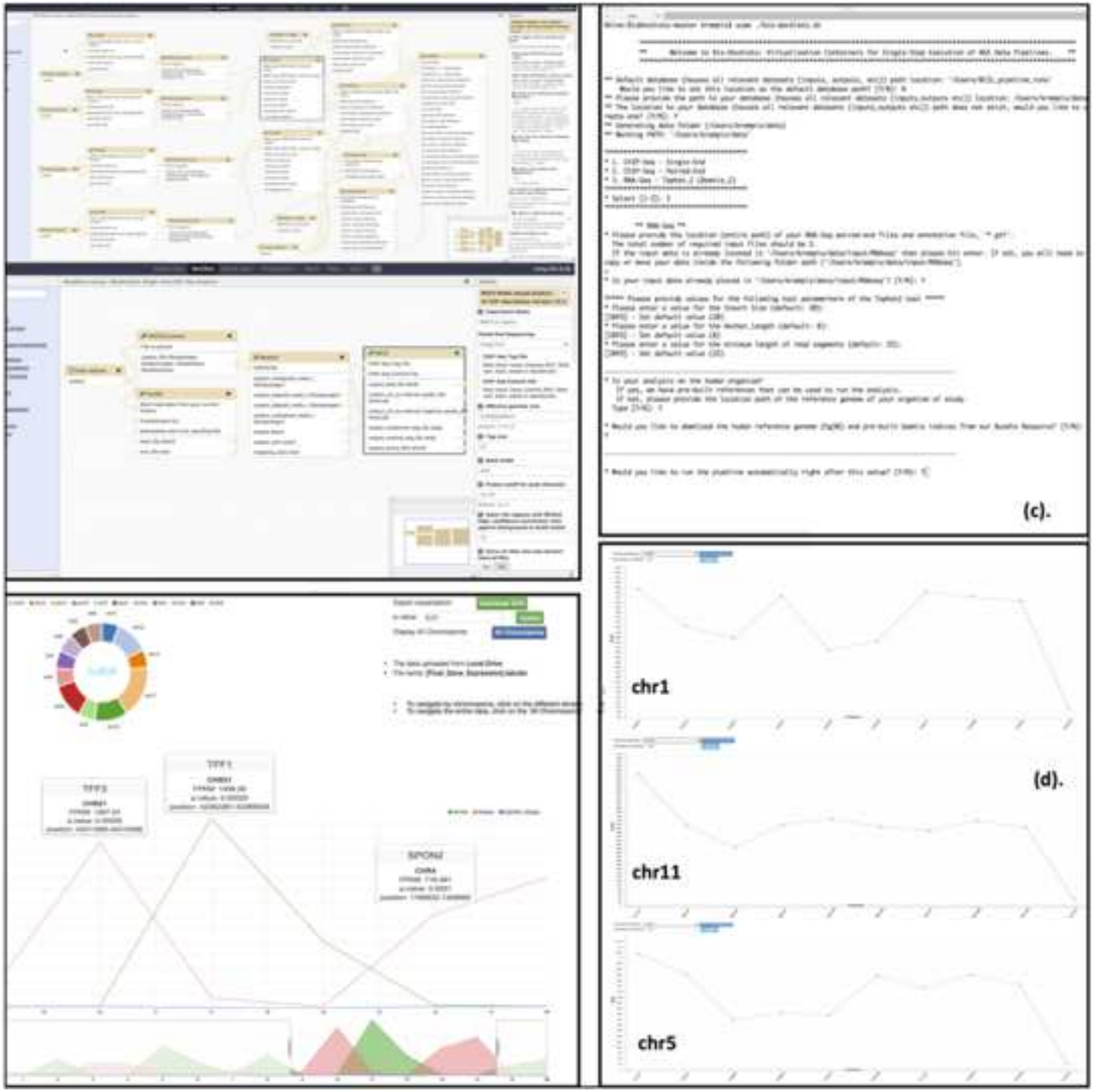

Click here to access/download
Supplementary Material
Supplemental_Final.pdf
