# Supplementary material for: Bio-Docklets: virtualization containers for single-step execution of NGS pipelines
Source: Gigascience. 2017 Jun 27;6(8):1–7. doi: 10.1093/gigascience/gix048 (PMC5569920; doi:10.1093/gigascience/gix048)

Bio-Docklets: Virtualization Containers for Single-Step Execution of NGS Pipelines.

Supplementary User Manual

Baekdoo Kim, Thahmina Ali, Carlos Lijeron, Enis Afgan and Konstantinos Krampis*
*Corresponding Author: kk104@hunter.cuny.edu

Contents

1 Prerequisites 1

2 How to run BioDocklets 1
- 2.1 Download BioDocklets script . . . 1
- 2.2 Access the Terminal . . . 1
- 2.3 Run BioDocklets . . . 1
- 2.4 Setting up BioDocklets . . . 1

3 How to use CloudLaunch to run BioDocklets 3
- 3.1 Find Biodocklets on CloudLaunch . . . 3
- 3.2 Logging In . . . 4
- 3.3 Setting up BioDocklets Appliance to Launch . . . 5
- 3.4 SSH into BioDocklets appliance . . . 9

4 BioDocklets additional attributes 10
- 4.1 Validating required input files . . . 10
- 4.2 Retrieving supporting data . . . 11
- 4.3 Accessing the pipeline . . . 12
- 4.4 Rerunning BioDocklets . . . 14
- 4.5 BioDocklets Run log . . . 14
- 4.6 BioDocklets Data Visualization . . . 15
- 4.7 BioDocklets Run Specifications . . . 15

5 Supplementary Figure 1 15

6 Supplementary Figure 2 16

1 Prerequisites

The Docker virtualization layer (https://docs.docker.com/engine/installation/) needs to be installed for the Linux or Mac computer (or Windows computer running a Linux virtual machine).

2 How to run BioDocklets

Video Tutorial: (https://tinyurl.com/run-BD)

2.1 Download BioDocklets script

Download the script from: https://github.com/BCIL/BioDocklets/archive/master.zip

2.2 Access the Terminal

Open the terminal in Linux (http://www.wikihow.com/Open-a-Terminal-Window-in-Ubuntu) or Mac (http://www.wikihow.com/Open-a-Terminal-Window-in-Mac).

2.3 Run BioDocklets

On a Mac the compressed master.zip file of the script needs to be unzipped (http://www.wikihow.com/Unzip-File#Mac_Default_Program_sub). Go in the directory where the script was saved and run it by using the following terminal commands:

$ cd ~/Downloads/BioDocklets-master/ (adjust to your download directory)
$ bash bio-docklets.sh

2.4 Setting up BioDocklets

Users can then follow the on-screen prompts, for providing the parameters for the data directories, selecting the pipeline to run, and additional options as shown in the following screenshots.

Select which pipeline to run:

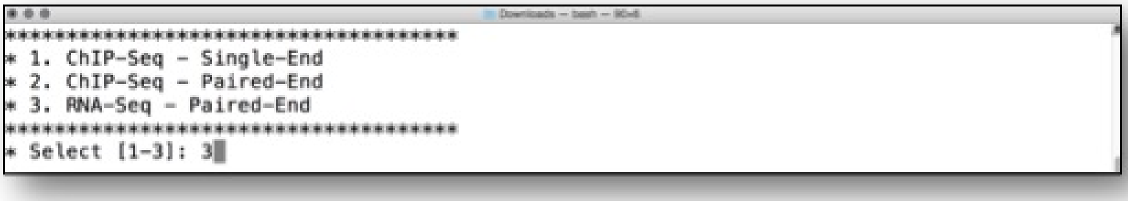

Set the default working directory (/Users/BCIL_pipeline_runs/) by providing the location of your inputs which will get transferred into the working directory location. (Mac instructions to find that location: http://www.wikihow.com/Find-the-Library-Folder-on-a-Mac):

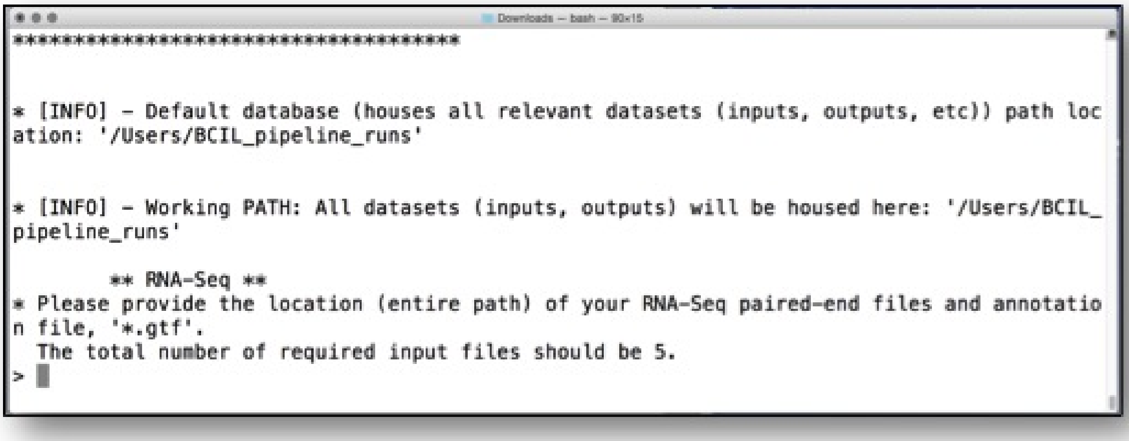

```
**************************************

* [INFO] - Default database (houses all relevant datasets (inputs, outputs, etc)) path loc
ation: '/Users/BCIL_pipeline_runs'

* [INFO] - Working PATH: All datasets (inputs, outputs) will be housed here: '/Users/BCIL_
pipeline_runs'

        ** RNA-Seq **
* Please provide the location (entire path) of your RNA-Seq paired-end files and annotatio
n file, '*.gtf'.
  The total number of required input files should be 5.
>
```

Specify parameters for the tools in the pipeline. More information about the tools options is explained in Suppl. Figure 1:

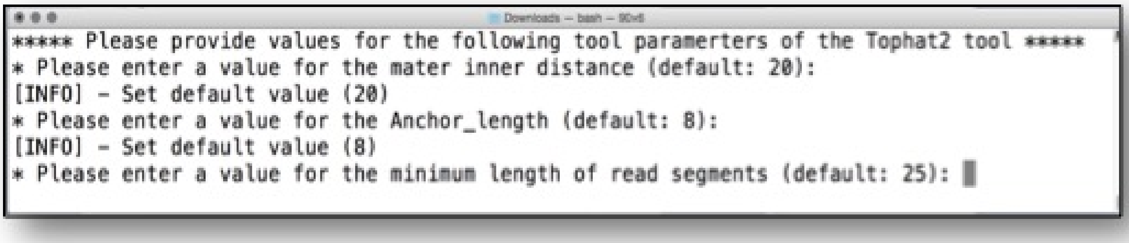

```
***** Please provide values for the following tool paramerters of the Tophat2 tool *****
* Please enter a value for the mater inner distance (default: 20):
[INFO] - Set default value (20)
* Please enter a value for the Anchor_length (default: 8):
[INFO] - Set default value (8)
* Please enter a value for the minimum length of read segments (default: 25):
```

Specify a known port that is available, if not the script will set one up. IMPORTANT: If BioDocklets is being run on CloudLaunch, then the following ports of either 8090 or 9010 must be entered. At most only two pipelines can be run on the same cloud server on CloudLaunch due to the availability of two ports: 8090, 9010.

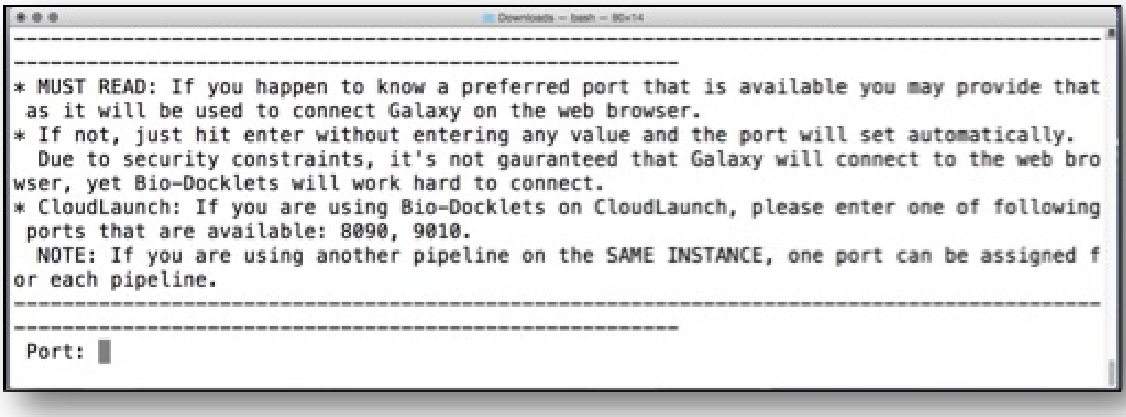

3 How to use CloudLaunch to run BioDocklets

Video Tutorial: (http://tinyurl.com/BD-cloud-launch)

3.1 Find Biodocklets on CloudLaunch

Go to: https://beta.launch.usegalaxy.org/marketplace and select the BioDocklet appliance to launch:

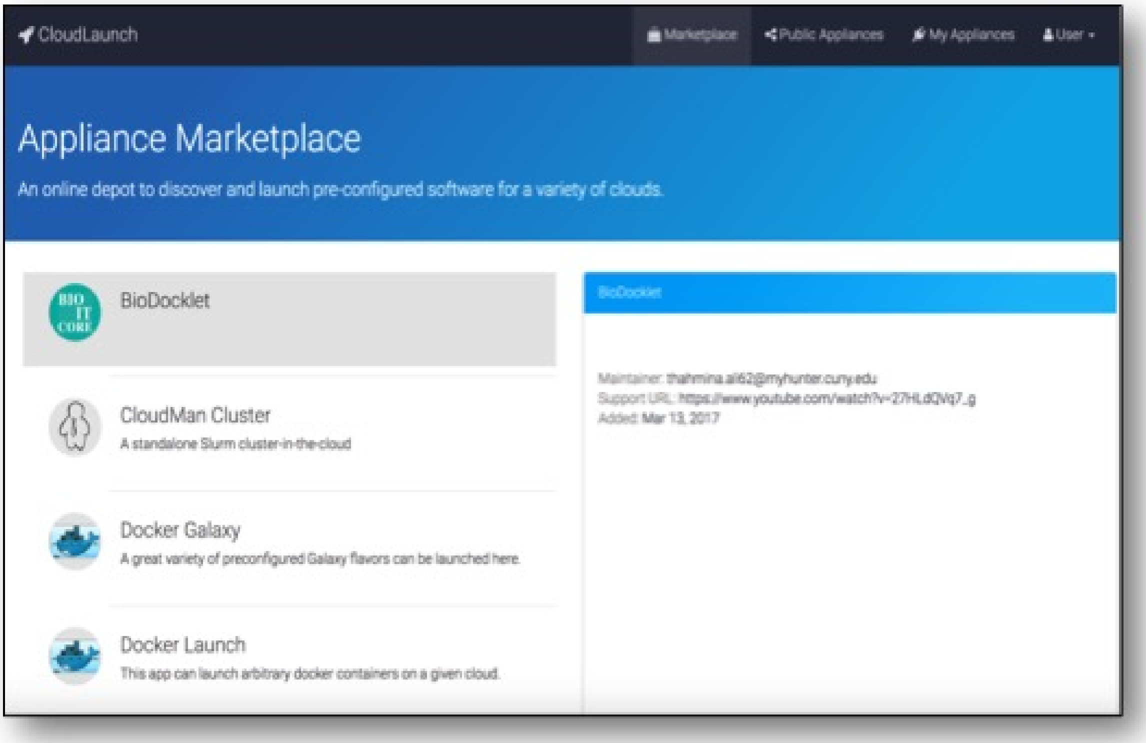

3.2 Logging In

A log in is required and can be done with any of the following account credentials:

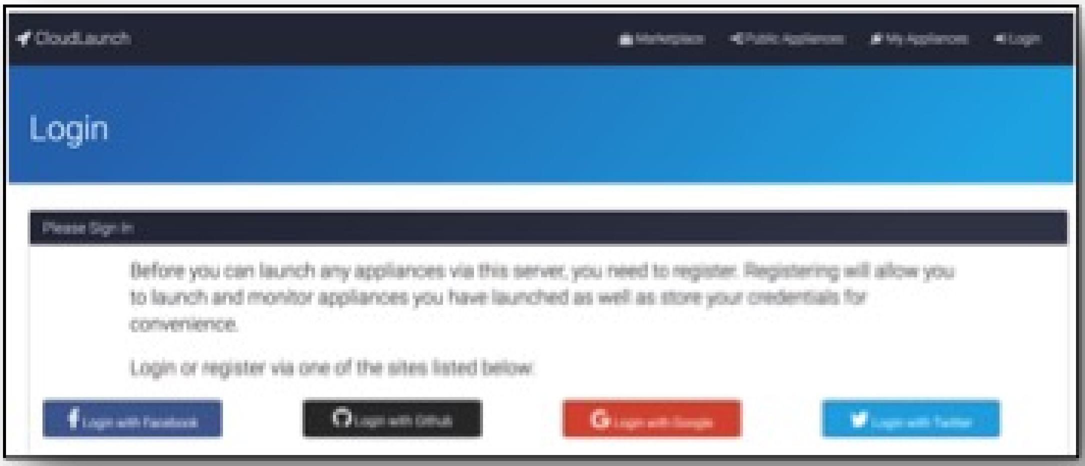

3.3 Setting up BioDocklets Appliance to Launch

The Biodocklet pipelines RNAseq and ChIPseq are offered as options and can be launched on the Amazon Web Services (AWS) cloud service. An access key ID and secret key is required from your AWS account, instructions to do so can be found here: http://tinyurl.com/create-aws-key After choosing a pipeline to launch and entering information about your AWS key credentials, click NEXT to continue:

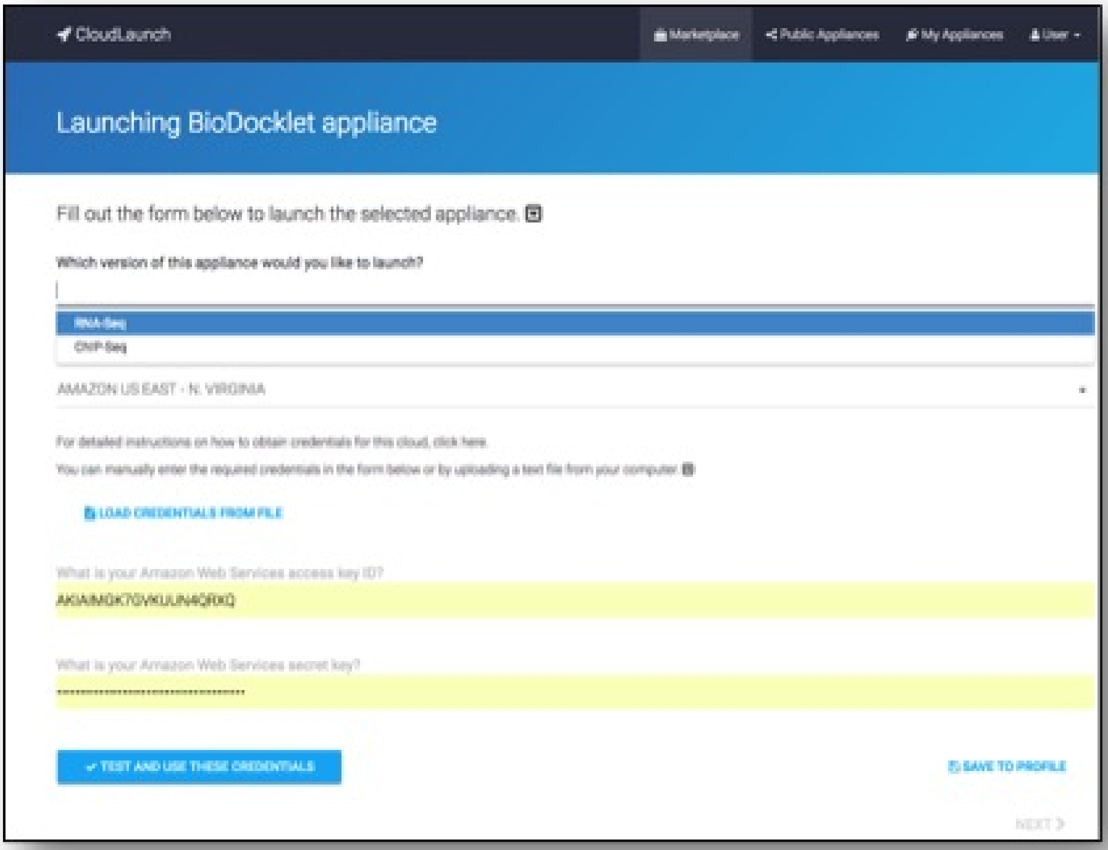

Choose a hardware (https://aws.amazon.com/ec2/instance-types/) for the AWS cloud server and enable Advanced cloud launch option:

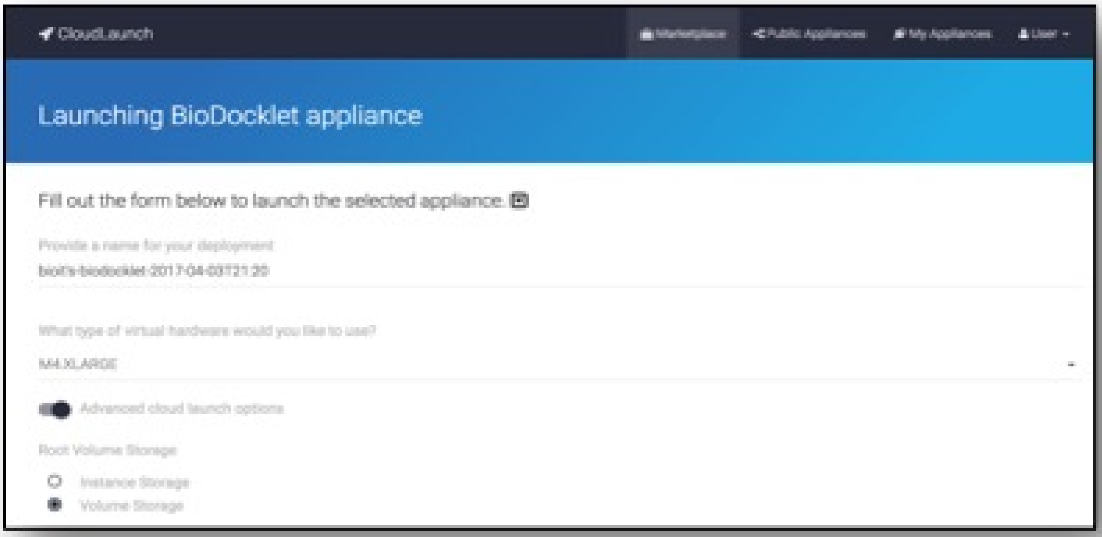

Select volume storage and enter a storage space amount (GB). A keypair can be selected or the default youre your AWS account thats available will be used. The pem file thats associated with the keypair must be made available as it will be used in the later steps to connect. A static IP address must also be selected, which will need to be allocated from your AWS account. Note: A static IP address dedicated for every biodocklet appliance that gets launched. When all specifications are entered click LAUNCH to proceed:

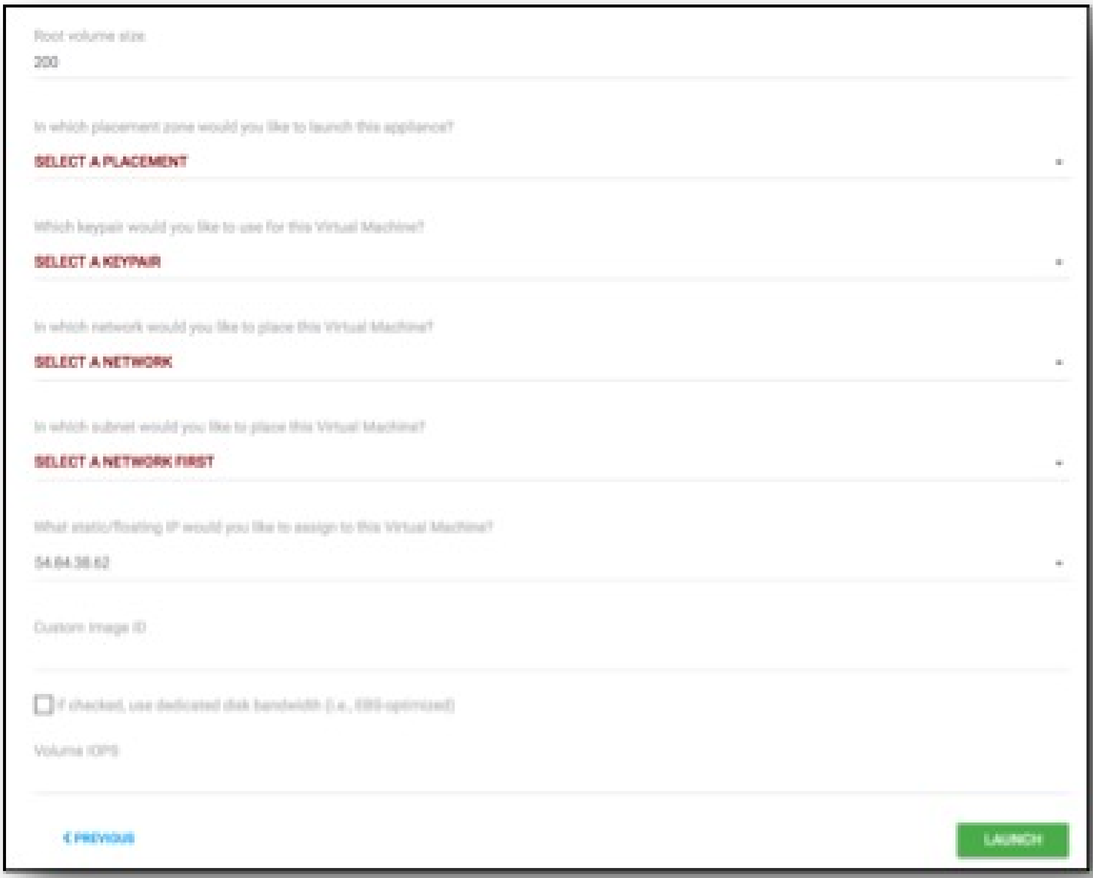

You will be redirected to the My Appliances that shows the status of the launch and when its ready to be used. Note: You cannot shut down or delete any instances from this page but need to use the cloud service provider interface to do so and even after an appliance has been shut down, its access URL will remain visible on this page:

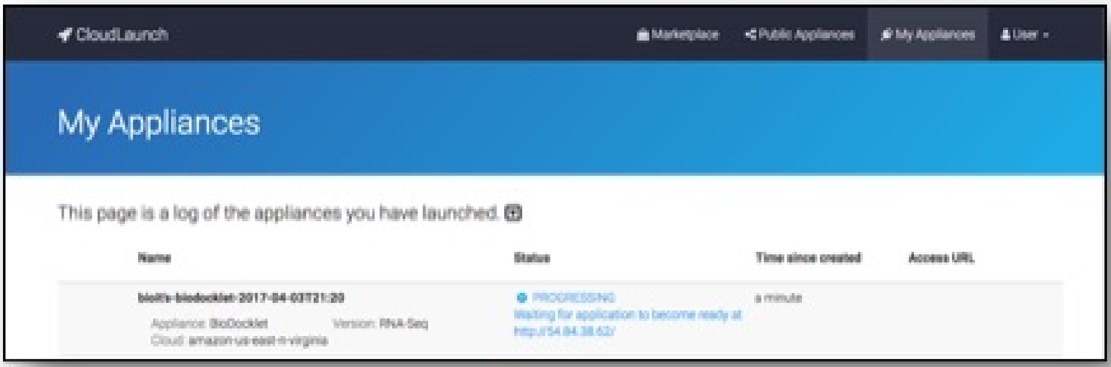

3.4 SSH into BioDocklets appliance

On your machine, open up the terminal and using the key pair pem file thats associated with the key pair selected in step 3 ssh into the appliance via using ubuntu as the username and the static IP address that was provided. Note: A warning message might occur:

```
bash-3.2# ssh -i kp.txt ubuntu@54.84.38.62
@@@@@@@@@@@@@@@@@@@@@@@@@@@@@@@@@@@@@@@@@@@@@@@@@@@@@@@@@@@
@    WARNING: REMOTE HOST IDENTIFICATION HAS CHANGED!     @
@@@@@@@@@@@@@@@@@@@@@@@@@@@@@@@@@@@@@@@@@@@@@@@@@@@@@@@@@@@
IT IS POSSIBLE THAT SOMEONE IS DOING SOMETHING NASTY!
Someone could be eavesdropping on you right now (man-in-the-middle attack)!
It is also possible that a host key has just been changed.
The fingerprint for the RSA key sent by the remote host is
17:85:0c:5c:e9:06:c3:63:00:28:71:81:a4:9f:dc:51.
Please contact your system administrator.
Add correct host key in /var/root/.ssh/known_hosts to get rid of this message.
Offending RSA key in /var/root/.ssh/known_hosts:7
RSA host key for 54.84.38.62 has changed and you have requested strict checking.
Host key verification failed.
```

To get around the warning message enter the following:

```
bash-3.2# ssh-keygen -R 54.84.38.62
# Host 54.84.38.62 found: line 7 type RSA
/var/root/.ssh/known_hosts updated.
Original contents retained as /var/root/.ssh/known_hosts.old
```

Go back and ssh again and you should be able to connect:

```
bash-3.2# ssh -i kp.txt ubuntu@54.84.38.62
The authenticity of host '54.84.38.62 (54.84.38.62)' can't be established.
RSA key fingerprint is 17:85:0c:5c:e9:06:c3:63:00:28:71:81:a4:9f:dc:51.
Are you sure you want to continue connecting (yes/no)? yes
Warning: Permanently added '54.84.38.62' (RSA) to the list of known hosts.
Welcome to Ubuntu 16.04.1 LTS (GNU/Linux 4.4.0-57-generic x86_64)

 * Documentation:  https://help.ubuntu.com
 * Management:     https://landscape.canonical.com
 * Support:        https://ubuntu.com/advantage

  Get cloud support with Ubuntu Advantage Cloud Guest:
    http://www.ubuntu.com/business/services/cloud

70 packages can be updated.
0 updates are security updates.


*** System restart required ***
Last login: Wed Apr  5 15:36:42 2017 from 146.95.231.49
ubuntu@ip-10-0-0-28:~$
```

To continue the rest follow go to the section How to run Biodocklets and follow the instructions and tutorial.

4 BioDocklets additional attributes

4.1 Validating required input files

One or two .FASTQ read files are required for single or paired-end CHIPseq respectively; Four .FASTQ input files are required for RNAseq, two forward/reverse paired end reads for treatment A and two for treatment B. In addition, the .gtf annotation file (http://useast.ensembl.org/info/data/ftp/index.html) has to be provided for non-human

organisms.

The script verifies that the right input data have been provided by the user.

```
== Warning! The provided path (/Users/thahminaali/Desktop/RNAseq/RNAseq) does not contain
fastq file(s).
The RNA-Seq pipeline requires four FASTQ format file(s) (*.fastq) and one GTF file (*.gtf)
. Below are more detail(s) of the path:
------------------------------------------------------------------------------------------
-----------------
  chr19_gene_annotation.gtf
------------------------------------------------------------------------------------------
-----------------

* Please provide the location (entire path) of your RNA-Seq paired-end files and annotatio
n file, '*.gtf'.
  The total number of required input files should be 5.
> 
```

The script also ensures to transfer the input datasets to the working directory (/Users/BCIL_pipeline_runs):

```
== The database directory will be generated ('/Users/BCIL_pipeline_runs') to house all the
 required data (input, outputs etc). Your input data must be relocated there, would you li
ke to copy(SLOW) or move(FAST) your input data from its current location?
== Please type 'Y'(copy) or 'N'(move): 
```

4.2 Retrieving supporting data

Besides selecting the pipelines to run and setting the parameters, the script offers the option to automatically download our pre-compiled indexes for the Human genome (used by the Bowtie / TopHat aligner for matching reads to the reference genome). The Human index download can take significant amount of time, depending on the network bandwidth.

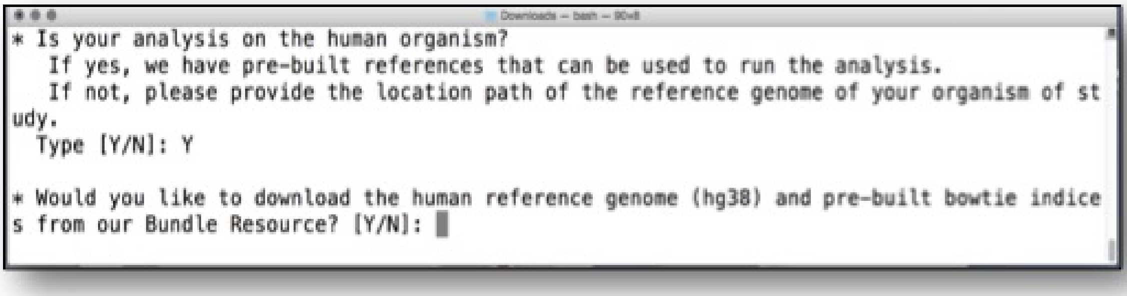

For alternative genomes users can add indexes manually, and pre-compiled indexes are available on the Bowtie website (http://bowtie-bio.sourceforge.net), with instructions how to compile an index for a custom genome, or the reference genome can be provided and the script will compile the indices for the preferred genome.

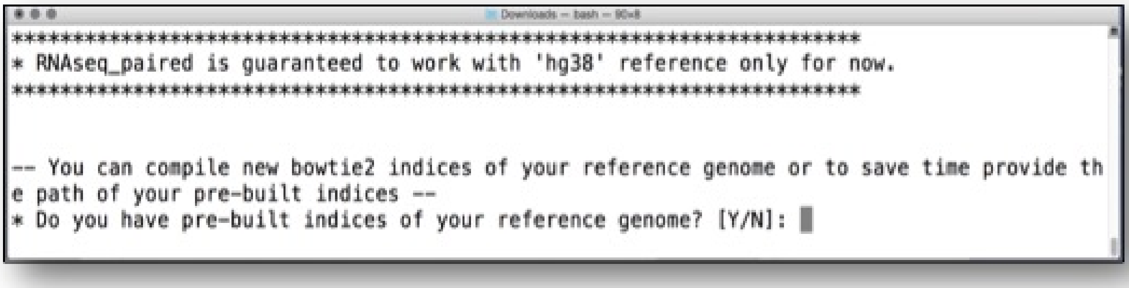

4.3 Accessing the pipeline

Users can view and edit the pipelines through the Galaxy workflow canvas, as the script will provide a web address under 'Galaxy server address,' which can be copied in a web browser.

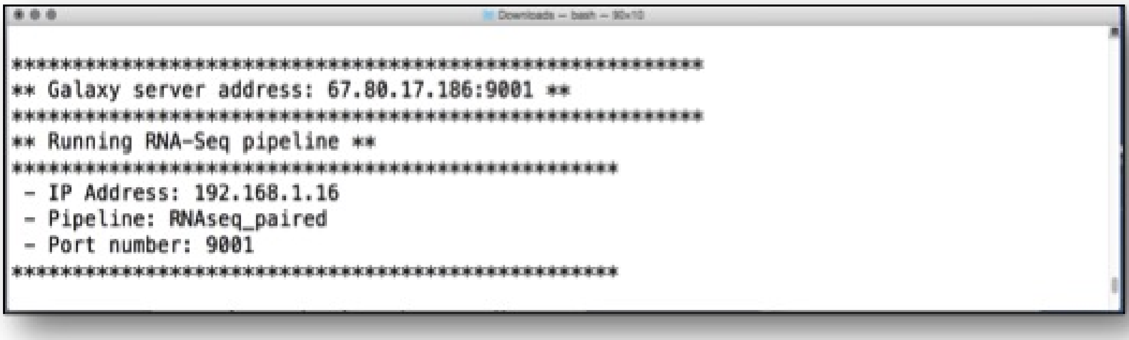

The following credentials are required to log in to the Galaxy server for access to the pipeline: username: user@galaxy.edu, password: biodocklet (no caps, no spaces):

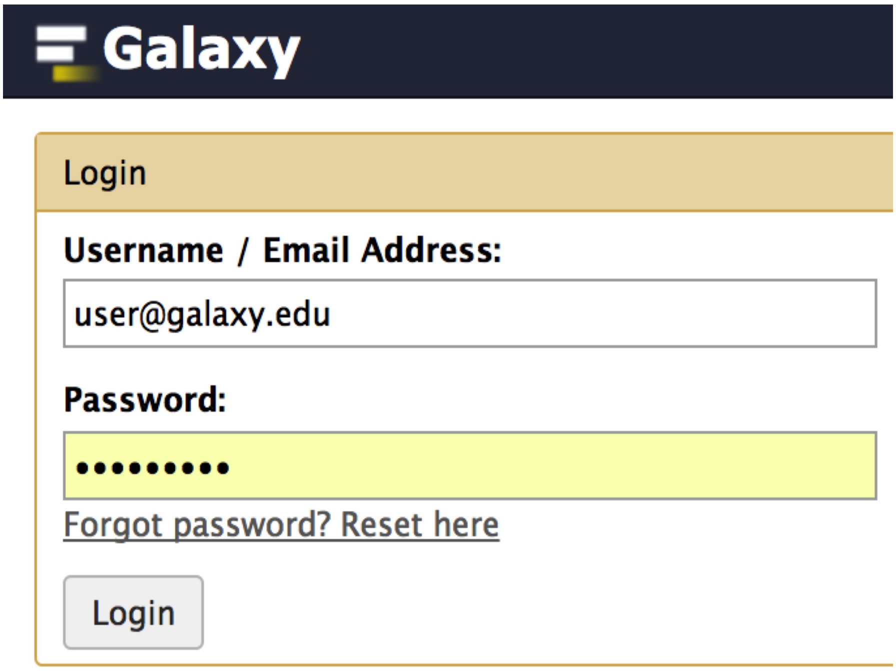

Once logged in, users can find the pipeline under 'Workflow' on the navigation panel. By clicking on the drop down arrow of the workflow, multiple options are offered and by selecting 'Edit' the pipeline and all of of its components can be viewed on the workflow canvas.

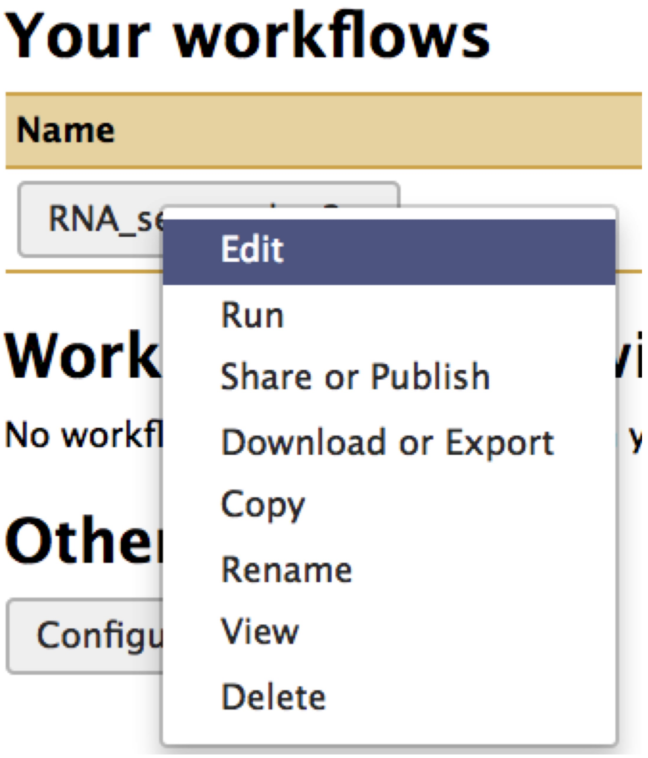

4.4 Rerunning BioDocklets

The script can be run as many times as the user desires, creating a different output directory for every run, and automatically detecting whether the genome indexes have been already downloaded or copied in the directory.

4.5 BioDocklets Run log

The script logs all activity and will notify the users once the processing has completed, and will terminate the Docker containers to release the computational resources. There is no need to move the data out of the output directory, as a new one is created for each run (along with a timestamp for the run in the name of the directory). This also enables running multiple instances of the pipelines / containers.

4.6 BioDocklets Data Visualization

When the pipeline completes, there will be multiple intermediate files in the output directory, but most users will prefer to open the single .html file which contains the CHIPseq or RNAseq visualization.

4.7 BioDocklets Run Specifications

The pipeline run times will be different based on the computing capacity of the host computer and size of input datasets. We recommend enough RAM memory (at least 8GB) and disk storage (at least 500GB for multiple runs), and minimum 4 CPU cores using a chipset such as the latest Intel i5 or i7.

5 Supplementary Figure 1

Algorithm parameters for the Tophat2 tool in the RNAseq pipeline and Bowtie2 and MACS2 tools in the ChIPseq pipeline.

RNAseq: Tophat2

Note: Mean Inner Distance option applies only to paired-end dataset pipeline.

Parameter	Function
Mean Inner Distance	inner distance betweeen reads
Anchor length (at least 3)	introns extending into exons
Minimum length of read segments	Each read is cut up into segments, each at least this long. These segments are mapped independently

ChIPseq: Bowtie2

Note: Option only applied to paired-end dataset pipeline.

Parameter	Function
Set the maximum fragment length for valid paired-end alignments	Determine if a concordant alignment exists

ChIPseq: MACS2

Parameter	Function
Effective genome size	genome size of organism of study
Band width	size of the window for model building
p-value cutoff for peak detection	calculated probability

6 Supplementary Figure 2

BioDocklets Interface Modalities: Non-Graphical (Terminal) vs Graphical (GUI)

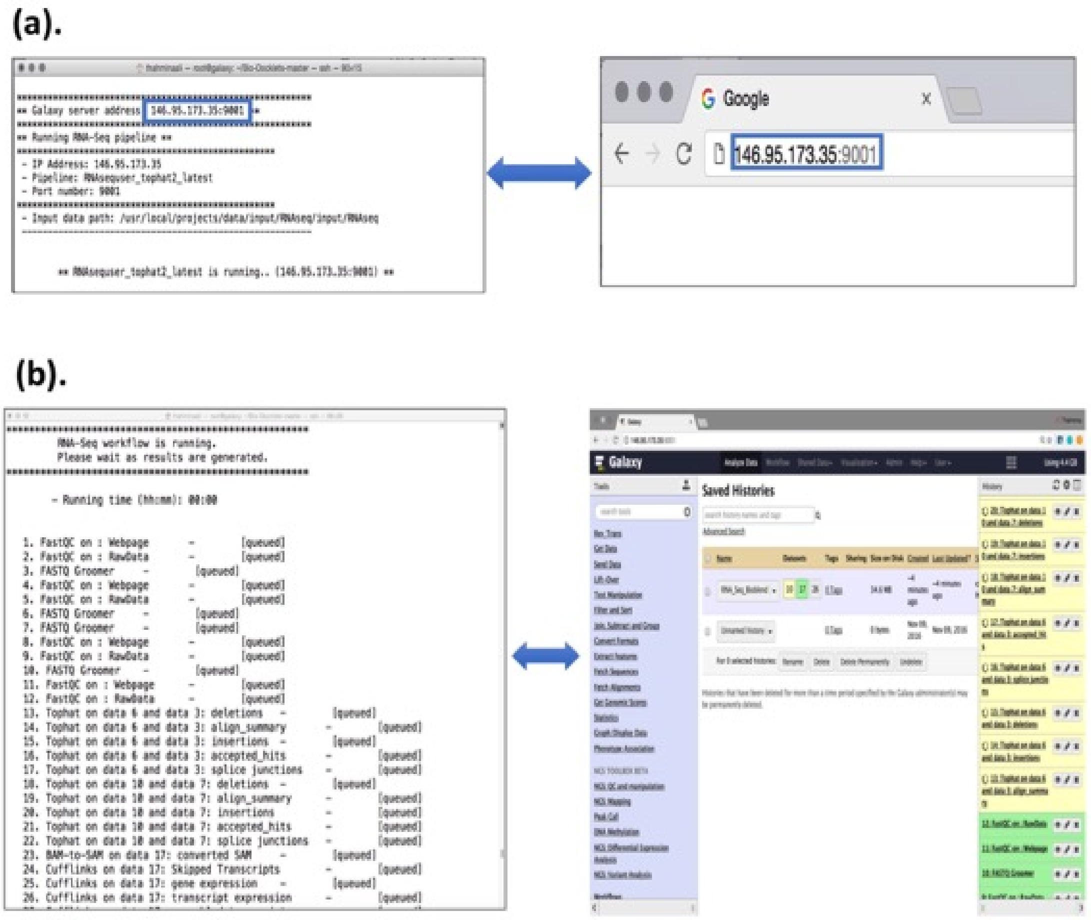

Supplement: Supplemental material [file gix048_Supp.pdf]
