# Supplementary material for: Bio-Docklets: virtualization containers for single-step execution of NGS pipelines
Source: Gigascience. 2017 Jun 27;6(8):1–7. doi: 10.1093/gigascience/gix048 (PMC5569920; doi:10.1093/gigascience/gix048)
Supplement: Response_to_Reviewer_Comments_Revision_1.pdf [file gix048_Response_to_Reviewer_Comments_Revision_1.pdf]

# Author's Response To Reviewer Comments

We have updated the following sections as requested by the editor:

Availability of data and materials

Snapshots of the supporting code are available from the GigaScience GigaDB repository [40].

Availability of supporting source code and requirements

Project name: Bio-Docklets
Project home page: https://github.com/BCIL/BioDocklets
DockerHub: https://hub.docker.com/r/bcil/biodocklets/tags/
Operating systems: Ubuntu and MacOS.
Programming language: python, shell
Other requirements: Docker virtualization layer needs to be installed for Linux or Mac
License: MIT

Added reference:

40. Kim B; Ali T; Lijeron C; Afgan E; Krampis K (2017): Supporting data for "Bio-Docklets: Virtualization Containers for Single-Step Execution of NGS Pipelines." GigaScience Database.
http://dx.doi.org/10.5524/100323

Response to reviewers:

Reviewer #1

1. Bio-Docklets are one approach to encapsulate bioinformatics tools and tool-chains in Docker containers. As such, they ease the installation and execution of bioinformatics tools, here with a strong emphasis on tool-chains (or workflows) in Galaxy. As a proof of principle, the authors ship two analysis pipelines - RNAseq and CHIPseq - as Bio-Docklets, accompanied by a driver script to execute them on any Docker-capable system, and demonstrate performance and ease of use. The supplementary user manual is largely excellent, but it remains a bit unclear if and how existing Bio-Docklet workflows can be extended (or how one creates an entirely new workflow within the framework).

On page 13 of the supplementary material we included the following information in respect to this: "users can paste on their web browser the IP address provided by the meta-script, in order to access the Galaxy workflow canvas (https://galaxyproject.org/learn/advanced-workflow/basic-editing/) for viewing or modifying either the RNAseq or CHIPseq workflow, or even creating a new workflow from scratch using the canvas. Users can then simply commit the modified container on DockerHub in order to share with others. Further extensibility in the case of implementing a new workflow, requires addition to the launch-pipeline.sh script of execution option for the new pipeline (https://github.com/BCIL/BioDocklets/blob/master/launch-pipeline.py, elif statement).

2. Other than that, I congratulate the authors on their work! I found the manuscript to be light reading, yet compelling (and relevant) enough to closely follow. I see Bio-Docklets value, share most of the authors' statements, and congratulate them on a well-written case study highlighting the potential and benefits of using Docker for reproducible research. I believe that the paper fits nicely into GigaScience's collection and scope, and recommend to accept it for publication after a minor revision.
The authors really appreciate the extensive and detailed review, support and kind comments of the reviewer.

3. Full disclosure: I'm a co-creator of bioboxes, a somewhat related approach but with a different scope and aim. I don't see bioboxes and Bio-Docklets competing, but rather imagine these playing along nicely. In theory, Bio-Docklets could internally even use bioboxes and thus benefit from a standardized interfaces for certain tool families (this is merely a remark to illustrate my point, no suggestion to the authors). I've read the Bio-Docklets preprint on bioRxiv, which seems to be largely identical with the submitted version, and thus were familiar with the concept already. Unfortunately, the manuscript version provided for review does not include page numbers (and the line numbers are poorly aligned); I start page numbering with 1 on the title page w/ title and authors, i.e. after the initial table inserted by the editorial system:

Page numbers have been inserted in the manuscript for improving the readability and helping the reviewers.

4. p2.22: I hate to be that guy, but Bremges et al., GigaScience 2015 (https://doi.org/10.1186/s13742-015-0087-0), would be a nice addition to the introduction to further highlight the use of Docker in "many recent studies", adding an applied and fully reproducible research study to the already cited benchmarking and packaging efforts.
The reference has been added, the authors realize that this should have been included from the start!

5. p2.27: Ref [5] is wrong, the primary publication for Galaxy is Afgan et al., NAR 2016, see:

https://galaxyproject.org/citing-galaxy/

The reference has been corrected (note that this is [6] now, as with the insertion of the Bremges et al. reference in the beginning of the manuscript, every reference has shifted by plus one).

6. p3.13: I do not understand why "the containers [...] scale up the analysis up to 200 million reads". The containers add very little overhead and therefore analysis volume is limited by (1) method choice and (2) available hardware, i.e. the server size; in theory it should be possible to analyze billions++ of reads.

We updated this specific sentence in the paper to read as following: "While the MiSeq instrument produces approximately 35 million reads, in the present study we tested our pipelines with data sets up to 200 million reads using public datasets, and given the minimal overhead of the Docker containers by simply using a larger capacity compute server users should be able to analyze multiples of that data size."

7. p3.34: I struggle with the wording of "we observed" and "we found", because the authors - from my understanding - exemplarily reproduced certain key findings from previous studies [16,17] and did not generate new insights. I'd highlight the replication fact here make it perfectly clear (instead of wording/framing it as results).

The authors agree with the reviewer regarding this omission. We have updated the sentence to read as: "In both cases, the CHIPseq output contained the same peaks (p-value < .001) on chromosomes 1, 4, 5, 7, 8, 11, 16 and 19, which harbor histone interactions with active role to tumor genesis found in earlier studies [17], and similarly for RNAseq regarding the differentially expressed genes that are active regulators in cancer progression [18]. "

8. p3.35: By "significant peaks" I assume the authors mean "peaks of statistical significance" (or smooth. along the line). The authors state a p-value treshold, but do not say which statistical test they actually performed.

Updated in the manuscript, p<0.01

9. p3.35: It might be of interest to add 2 supplementary tables summarizing the results: (1) peaks with their genomic position, and (2) differentially expressed genes. I realize that this is not the main scope nor new (?), but if findings are explicitly mentioned in the text, they should be backed up with data.

The complete list of results for the significant peaks or differentially expressed genes for the chromosomal locations shown in the figures of the manuscript, have been included as supplementary data.

10. p3.42: "Beyond availability for [...] the Amazon cloud" It is not clear why the Bio-Docklets are limited to AWS, in theory they should work on any cloud infrastructure (as long as Docker is available) - right?

We have added the following text to this section of the manuscript: "Furthermore, Bio-Dockets can be executed by running the meta-script on any computing platform such as the Linux servers on Amazon and Google compute clouds, or a local compute server that has Docker pre-installed, or installed during the first run of the script by providing and administrative password."

11. p4.Table 1: I personally think the table would read better if it were transposed, i.e. if rows and columns were switched; I guess it comes down to personal preferences.

The authors have tried both orientations before submitting the manuscript. We have settled in the current orientation, because2 vertical columns fit better in the manuscript page. Furthermore, the long lines with the dataset information and URLs, become cluttered in reverse orientation from the one the table has been entered in the manuscript.

12. p5.40: "HTML / Javascript-D3" Please state which HTML version (I assume HTML5) and use the term D3, Data-Driven Documents, or simply D3.js instead of Javascript-D3 (change in the reference list, too), see: https://github.com/d3/d3/wiki

Updated in the manuscript and the references section, to reflect HTML5 and D3.js.

13. p5.45: I'd use "vectorized SVG" instead of "high-resolution SVG" (also in Fig. 1's legend on p6)

Updated in the manuscript.

14. p6.Fig 1: Changed order: "The Bio-Docklets environment with (a). an interactive [...]"

Updated in the manuscript.

15. p8.11: It's "bioboxes", not "BioBoxes".

Updated throughout the manuscript.

16. p8.15: "while [bioboxes] at the time of our study included a total number of 8 containers" This is not correct, I count at least 26 containers here: https://hub.docker.com/u/bioboxes/, with more bioboxes available as result of the CAMI challenge (Sczyrba et al., bioRxiv 2017). I have no idea why the CAMI-bioboxes are not on DockerHub, yet, but nevertheless: 26 publicly available on DockerHub, and dozens more within CAMI (in fact, CAMI and nucleotid.es were the main motivation and purpose to create the bioboxes concept).

We have updated the manuscript to reflect the information of 26 bioboxes containers (we have mistakenly counted 8 containers from the list at http://bioboxes.org/available-bioboxes/, and missed looking at DockerHub – we thank the reviewer for that crucial correction).

17. p8.25: I do not agree that "[bioboxes] provide [...] no user interaction or other options for using the containers are provided" is a bad thing per se, but rather desired: bioboxes hide any expert parameters from the user, the author of a biobox should take care of appropriate parameter selection - either by empirically testing within the biobox, or by sensible defaults. This is a similar argument to one given by the authors further down (p8.55) that Bio-Docklets "essentially provides bioinformatics black-boxes that expose a single input and output endpoint, while internally [...]". Please reword the initial half-sentence if you agree.

The authors appreciate the reviewer's suggestion and we have reworded the section as following:
"Along the same lines, bioboxes provide a standardized interface where users can run bioinformatics tools and specify data directories with a single command, in addition to a novel framework for standardizing bioinformatics tool deployment in containers. While there is no user interaction or options for a workflow engine or multi-step pipeline capabilities, the author of a biobox empirically pre-selects the appropriate parameters during implementation, and similar to Bio-Docklets abstracts all the details from the users in order to standardize and streamline bioinformatics analysis."

18. p8.37: Ref [31] is wrong, instead of the URL the primary publication for CytoScape is either Shannon et al., Genome Res. 2003, or Cline et al., Nat Protoc. 2007, see: http://wiki.cytoscape.org/Publications
Corrected.

19. p 9.10: Ref [34] is outdated, NextFlow was recently published as: Di Tomasso et al., Nat Methods 2017, https://doi.org/10.1038/nbt.3820

Corrected, this is a great paper indeed!

20. discussion: I encourage the authors to look at CWL, http://www.commonwl.org/, and (briefly) discuss if and how CWL and Bio-Docklets can come together. CWL seems to gain a lot of traction recently and should be included.

We have added a paragraph on CWL in the manuscript that reads as following: "The Common Workflow Language (CWL) offers a flexible solution for composing and sharing data analysis workflows but, for the time being at least, it is focused on the bioinformaticians composing workflows as text files – a task not aimed at biology researchers and non-technical experts. Currently, there is no official repository of existing CWL workflows, although several instances of developed workflows can be found at online source code repositories. Importantly, those repositories are not vetted or based on pipelines published after peer review, which is the case for the pipelines made available via Bio-Docklets. Finally, executing a CWL workflow requires a CWL runtime environment on the user's system, whose setup may, again, represent a challenge for a biologist".

21. references: Please add access times to all URLs

We have re-visited all the URLs in order to verify at the time of the present writing (June 2017), and noted that in the beginning of the references section.

22. general: "Bio-Docklets" throughout the manuscript and in the title of the manual vs. "BioDocklets" in the manual itself, I'd pick either version and use it consistently.

We have updated the manuscript and supplementary manual to use consistently "BioDocklets" , which is more readable than "Bio-Docklets".

Reviewer #2:

1. The authors describe Bio-Docklets, a novel method to consolidate Galaxy based workflows by using Docker container technology and providing the ability to programmatically interact with such environment. This is a study relevant to researchers interested in developing reproducible pipelines.

We thank the reviewer for the time taken to read and provide positive feedback to the manuscript.

2. The manuscript is well written though there are a few typos to correct: page 3 line 43, "users anyone"; page 8, line 46, "user-freindly" and later "and should they require to modify the pipelines .." (it doesn't read well).

These typos have been corrected in the manuscript, and the sentence updated as "In contrast, with our approach a researcher can easily access the rich, user-friendly interface of the Galaxy workflow canvas to easily modify or extend our pipelines, through substituting the existing tools or adding new ones from the Galaxy ToolShed. "

3. The authors have properly discussed their method comparing it with other workflows containerisation approaches proposed by the community, however they do not mention that the Galaxy platform provides a built-in integration for Docker containers (1). The authors should discuss advantages and disadvantages of their approach over the one provided natively by the Galaxy platform.

We have added the following text in the discussion section of the manuscript discussing the integration of Galaxy with Docker and how it compares with our approach: "The Galaxy platform provides an option to execute containerized tools as computational jobs [https://galaxyproject.org/admin/tools/docker/] on a local cluster or the cloud, allowing developers and system administrators to tap on the plethora of containers with pre-configured tools in order to customize and enhance the functionality of a Galaxy installation. However, this requires modifying Galaxy configuration files in addition to setting up Docker [https://github.com/apetkau/galaxy-hackathon-2014/tree/master/smalt], on each installation separately. Our approach instead is targeted at users without the technical expertise to administer Galaxy or configure Docker, by automating the setup of both components using a single meta-script. Furthermore, our goal is to provide an integrated solution with pre-configured data analysis pipelines, which can be deployed across systems ranging from single compute servers used in a laboratory, to a cluster or the cloud. We realize that with the availability of Galaxy instances in Docker containers and VirtualBox machines [8], Galaxy community developers can implement solutions that provide automated deployment of all components with a similar approach to our meta-script. For example, code could be implemented that would first deploy a virtualized Galaxy server that is customized to use Docker as its job execution environment, and this code could additionally retrieve containers with bioinformatics tools from DockerHub or other repositories. This would provide a more broad, infrastructure deployment approach compared to ours, but would still require that developers provide a "wrapper" for new tools in order to become accessible for users through the Galaxy interface."

4. Moreover I believe that the manuscript will be strengthened if the authors provide a more balanced view on the possible limitations of the method proposed, for example regarding the scaling ability of their method. Is this possible to deploy the execution of a Bio-Docklets pipeline in a cluster of computers? If the method relies on the Galaxy subsystem for this, how the containerisation is managed in a distributed execution? Along the same lines, though this approach is powerful because it allows all the dependencies to be consolidated into a Docker image, some doubts arise on the ability to transparently replicate and track changes of a Bio-Docklets pipeline and how it can be deposited in a source code repository. Authors may want to provide some notes at this regard.

We have added in the manuscript the following text (quoted below) regarding scalability of Bio-Docklets. Furthermore, any changes on the meta-script and internal scripts driving Bio-Docklets will be committed to the project's repository on GitHub (https://github.com/BCIL/BioDocklets), in addition to the repository on DockerHub for the Bio-Docklets containers (https://hub.docker.com/r/bcil/biodocklets/).

"A significant advantage of Galaxy is scalability through the option for integration with a computer cluster in the back-end [https://galaxyproject.org/admin/config/performance/production-server/], enabling high throughput data analysis within a production environment. Since Bio-Docklets also include a fully-featured Galaxy instance, by editing the configuration files as described in they can similarly connect to a cluster. Furthermore on a computer server that has ample computational capacity, users can simply run the meta-script more than one times in order to start multiple instances of Bio-Docklets and process input datasets from different experiments in parallel. This is similar to multiple job submissions on the cluster of a typical high-throughput Galaxy installation, and despite the fact that a new Galaxy instance is started within each BioDocklet there is minimal computational overhead since the instance runs only one pipeline under a single user. The Docker containers have also very little overhead, and tools such as the read aligner or transcript assembler that process millions of reads in our bioinformatics pipelines essentially consume all the computational resources. An improvement for the future would be to add to our script an option for advanced cluster integration and through including DRMAA software libraries in our containers, for the script to auto-configure these libraries for computational job submission on a specific cluster. Another approach given that there is not limit on the resources that a Docker container utilizes, is to parallelize the pipelines internally assuming that the user has access to a powerful server to run the Bio-Docklets. While this would be feasible for tools performing independent tasks such as read alignment using for example the file split options in Galaxy pipeline composition, other tools such as genome assemblers are monolithic and the only option for scalability, is if they offer the option for multithreaded execution in the implementation of the algorithm"

5.The proper citation for Nextflow is doi:10.1038/nbt.3820.

The Nextflow citation has been updated to reflect the most recent article in Nature Biotechnology.

1) https://galaxyproject.org/admin/tools/docker/
