# Supplementary material for: Bio-Docklets: virtualization containers for single-step execution of NGS pipelines
Source: Gigascience. 2017 Jun 27;6(8):1–7. doi: 10.1093/gigascience/gix048 (PMC5569920; doi:10.1093/gigascience/gix048)
Supplement: Reviewer_1_Report_(Original_Submission).pdf [file gix048_Reviewer_1_Report_(Original_Submission).pdf]

Reviewer Report

Title: Bio-Docklets: Virtualization Containers for Single-Step Execution of NGS Pipelines.

Version: Original Submission **Date:** 4/27/2017

Reviewer name: Andreas Bremges

Reviewer Comments to Author:

Bio-Docklets are one approach to encapsulate bioinformatics tools and tool-chains in Docker containers. As such, they ease the installation and execution of bioinformatics tools, here with a strong emphasis on tool-chains (or workflows) in Galaxy. As a proof of principle, the authors ship two analysis pipelines - RNAseq and CHIPseq - as Bio-Docklets, accompanied by a driver script to execute them on any Docker-capable system, and demonstrate performance and ease of use. The supplementary user manual is largely excellent, but it remains a bit unclear if and how existing Bio-Docklet workflows can be extended (or how one creates an entirely new workflow within the framework). Other than that, I congratulate the authors on their work! I found the manuscript to be light reading, yet compelling (and relevant) enough to closely follow. I see Bio-Docklets value, share most of the authors' statements, and congratulate them on a well-written case study highlighting the potential and benefits of using Docker for reproducible research. I believe that the paper fits nicely into GigaScience's collection and scope, and recommend to accept it for publication after a minor revision.Full disclosure: I'm a co-creator of bioboxes, a somewhat related approach but with a different scope and aim. I don't see bioboxes and Bio-Docklets competing, but rather imagine these playing along nicely. In theory, Bio-Docklets could internally even use bioboxes and thus benefit from a standardized interfaces for certain tool families (this is merely a remark to illustrate my point, no suggestion to the authors). I've read the Bio-Docklets preprint on bioRxiv, which seems to be largely identical with the submitted version, and thus were familiar with the concept already.Unfortunately, the manuscript version provided for review does not include page numbers (and the line numbers are poorly aligned); I start page numbering with 1 on the title page w/ title and authors, i.e. after the initial table inserted by the editorial system:- p2.22: I hate to be that guy, but Bremges et al., GigaScience 2015 (https://doi.org/10.1186/s13742-015-0087-0), would be a nice addition to the introduction to further highlight the use of Docker in "many recent studies", adding an applied and fully reproducible research study to the already cited benchmarking and packaging efforts.- p2.27: Ref [5] is wrong, the primary publication for Galaxy is Afgan et al., NAR 2016, see: https://galaxyproject.org/citing-galaxy/- p3.13: I do not understand why "the containers [...] scale up the analysis up to 200 million reads". The containers add very little overhead and therefore analysis volume is limited by (1) method choice and (2) available hardware, i.e. the server size; in theory it should be possible to analyze billions++ of reads.- p3.34: I struggle with the wording of "we observed" and "we found", because the authors - from my understanding - exemplarily reproduced certain key findings from previous studies [16,17] and did not generate new insights. I'd highlight the replication fact here make it perfectly clear (instead of wording/framing it as results).- p3.35: By "significant peaks" I assume the authors mean "peaks of statistical significance" (or smth. along the line). The authors state a p-value treshold, but do not say which statistical test they actually performed.- p3.35: It might be of interest to

add 2 supplementary tables summarizing the results: (1) peaks with their genomic position, and (2) differentially expressed genes. I realize that this is not the main scope nor new (?), but if findings are explicitly mentioned in the text, they should be backed up with data.- p3.42: "Beyond availability for [...] the Amazon cloud" It is not clear why the Bio-Docklets are limited to AWS, in theory they should work on any cloud infrastructure (as long as Docker is available) - right?- p4.Table 1: I personally think the table would read better if it were transposed, i.e. if rows and columns were switched; I guess it comes down to personal preferences.- p5.40: "HTML / Javascript-D3" Please state which HTML version (I assume HTML5) and use the term D3, Data-Driven Documents, or simply D3.js instead of Javascript-D3 (change in the reference list, too), see: https://github.com/d3/d3/wiki- p5.45: I'd use "vectorized SVG" instead of "high-resolution SVG" (also in Fig. 1's legend on p6)- p6.Fig 1: Changed order: "The Bio-Docklets environment with (a). an interactive [...]"- p8.11: It's "bioboxes", not "BioBoxes".- p8.15: "while [bioboxes] at the time of our study included a total number of 8 containers" This is not correct, I count at least 26 containers here: https://hub.docker.com/u/bioboxes/, with more bioboxes available as result of the CAMI challenge (Sczyrba et al., bioRxiv 2017). I have no idea why the CAMI-bioboxes are not on DockerHub, yet, but nevertheless: 26 publicly available on DockerHub, and dozens more within CAMI (in fact, CAMI and nucleotid.es were the main motivation and purpose to create the bioboxes concept).- p8.25: I do not agree that "[bioboxes] provide [...] no user interaction or other options for using the containers are provided" is a bad thing per se, but rather desired: bioboxes hide any expert parameters from the user, the author of a biobox should take care of appropriate parameter selection - either by empirically testing within the biobox, or by sensible defaults. This is a similar argument to one given by the authors further down (p8.55) that Bio-Docklets "essentially provides bioinformatics black-boxes that expose a single input and output endpoint, while internally [...]". Please reword the initial half-sentence if you agree.- p8.37: Ref [31] is wrong, instead of the URL the primary publication for CytoScape is either Shannon et al., Genome Res. 2003, or Cline et al., Nat Protoc. 2007, see: http://wiki.cytoscape.org/Publications- p 9.10: Ref [34] is outdated, NextFlow was recently published as: Di Tomasso et al., Nat Methods 2017, https://doi.org/10.1038/nbt.3820- discussion: I encourage the authors to look at CWL, http://www.commonwl.org/, and (briefly) discuss if and how CWL and Bio-Docklets can come together. CWL seems to gain a lot of traction recently and should be included.- references: Please add access times to all URLs- general: "Bio-Docklets" throughout the manuscript and in the title of the manual vs. "BioDocklets" in the manual itself, I'd pick either version and use it consistently.I look forward to the revised version,Andreas Bremges

Level of Interest

Please indicate how interesting you found the manuscript: An article of importance in its field

Quality of Written English

Please indicate the quality of language in the manuscript: Acceptable

Declaration of Competing Interests

Please complete a declaration of competing interests, considering the following questions:

- Have you in the past five years received reimbursements, fees, funding, or salary from an organisation that may in any way gain or lose financially from the publication of this manuscript, either now or in the future?
- Do you hold any stocks or shares in an organisation that may in any way gain or lose financially from the publication of this manuscript, either now or in the future?
- Do you hold or are you currently applying for any patents relating to the content of the manuscript?
- Have you received reimbursements, fees, funding, or salary from an organization that holds or has applied for patents relating to the content of the manuscript?
- Do you have any other financial competing interests?
- Do you have any non-financial competing interests in relation to this paper?

If you can answer no to all of the above, write 'I declare that I have no competing interests' below. If your reply is yes to any, please give details below.

6. I'm a co-creator of bioboxes (i.e. standardized containers for interchangeable bioinformatics software). However, because bioboxes and Bio-Docklets have different scopes and goals, and therefore do not compete, I believe that I can review this paper without a conflict of interest or review bias.

I agree to the open peer review policy of the journal. I understand that my name will be included on my report to the authors and, if the manuscript is accepted for publication, my named report including any attachments I upload will be posted on the website along with the authors' responses. I agree for my report to be made available under an Open Access Creative Commons CC-BY license (http://creativecommons.org/licenses/by/4.0/). I understand that any comments which I do not wish to be included in my named report can be included as confidential comments to the editors, which will not be published.

I agree to the open peer review policy of the journal
