# Supplementary material for: Bio-Docklets: virtualization containers for single-step execution of NGS pipelines
Source: Gigascience. 2017 Jun 27;6(8):1–7. doi: 10.1093/gigascience/gix048 (PMC5569920; doi:10.1093/gigascience/gix048)
Supplement: Reviewer_2_Report_(Original_Submission).pdf [file gix048_Reviewer_2_Report_(Original_Submission).pdf]

Reviewer Report

Title: Bio-Docklets: Virtualization Containers for Single-Step Execution of NGS Pipelines.

Version: Original Submission **Date:** 5/9/2017

Reviewer name: Paolo Di Tommaso

Reviewer Comments to Author:

The authors describe Bio-Docklets, a novel method to consolidate Galaxy based workflows by using Docker container technology and providing the ability to programmatically interact with such environment. This is a study relevant to researchers interested in developing reproducible pipelines.The manuscript is well written though there are a few typos to correct: page 3 line 43, "users anyone"; page 8, line 46, "user-freindly" and later "and should they require to modify the pipelines .." (it doesn't read well).The authors have properly discussed their method comparing it with other workflows containerisation approaches proposed by the community, however they do not mention that the Galaxy platform provides a built-in integration for Docker containers (1). The authors should discuss advantages and disadvantages of their approach over the one provided natively by the Galaxy platform.Moreover I believe that the manuscript will be strengthened if the authors provide a more balanced view on the possible limitations of the method proposed, for example regarding the scaling ability of their method. Is this possible to deploy the execution of a Bio-Docklets pipeline in a cluster of computers? If the method relies on the Galaxy subsystem for this, how the containerisation is managed in a distributed execution? Along the same lines, though this approach is powerful because it allows all the dependencies to be consolidated into a Docker image, some doubts arise on the ability to transparently replicate and track changes of a Bio-Docklets pipeline and how it can be deposited in a source code repository. Authors may want to provide some notes at this regard.The proper citation for Nextflow is doi:10.1038/nbt.3820.1) https://galaxyproject.org/admin/tools/docker/

Level of Interest

Please indicate how interesting you found the manuscript: An article whose findings are important to those with closely related research interests

Quality of Written English

Please indicate the quality of language in the manuscript: Needs some language corrections before being published

Declaration of Competing Interests

Please complete a declaration of competing interests, considering the following questions:

- Have you in the past five years received reimbursements, fees, funding, or salary from an organisation that may in any way gain or lose financially from the publication of this manuscript, either now or in the future?
- Do you hold any stocks or shares in an organisation that may in any way gain or lose financially from the publication of this manuscript, either now or in the future?
- Do you hold or are you currently applying for any patents relating to the content of the manuscript?
- Have you received reimbursements, fees, funding, or salary from an organization that holds or has applied for patents relating to the content of the manuscript?
- Do you have any other financial competing interests?
- Do you have any non-financial competing interests in relation to this paper?

If you can answer no to all of the above, write 'I declare that I have no competing interests' below. If your reply is yes to any, please give details below.

I have no conflict of interests.

I agree to the open peer review policy of the journal. I understand that my name will be included on my report to the authors and, if the manuscript is accepted for publication, my named report including any attachments I upload will be posted on the website along with the authors' responses. I agree for my report to be made available under an Open Access Creative Commons CC-BY license (http://creativecommons.org/licenses/by/4.0/). I understand that any comments which I do not wish to be included in my named report can be included as confidential comments to the editors, which will not be published.

I agree to the open peer review policy of the journal
